# Network-level enrichment provides a framework for biological interpretation of machine learning results

**DOI:** 10.1162/netn_a_00383

**Published:** 2024-10-01

**Authors:** Jiaqi Li, Ari Segel, Xinyang Feng, Jiaxin Cindy Tu, Andy Eck, Kelsey T. King, Babatunde Adeyemo, Nicole R. Karcher, Likai Chen, Adam T. Eggebrecht, Muriah D. Wheelock

**Affiliations:** Department of Statistics and Data Science, Washington University in St. Louis, MO, USA; Mallinckrodt Institute of Radiology, Washington University in St. Louis, MO, USA; Department of Neurology, Washington University in St. Louis, MO, USA; Department of Psychiatry, Washington University in St. Louis, MO, USA

**Keywords:** Functional connectivity, Machine learning, Twins, Feature selection, Brain networks, HCP

## Abstract

Machine learning algorithms are increasingly being utilized to identify brain connectivity biomarkers linked to behavioral and clinical outcomes. However, research often prioritizes prediction accuracy at the expense of biological interpretability, and inconsistent implementation of ML methods may hinder model accuracy. To address this, our paper introduces a network-level enrichment approach, which integrates brain system organization in the context of connectome-wide statistical analysis to reveal network-level links between brain connectivity and behavior. To demonstrate the efficacy of this approach, we used linear support vector regression (LSVR) models to examine the relationship between resting-state functional connectivity networks and chronological age. We compared network-level associations based on raw LSVR weights to those produced from the forward and inverse models. Results indicated that not accounting for shared family variance inflated prediction performance, the k-best feature selection via Pearson correlation reduced accuracy and reliability, and raw LSVR model weights produced network-level associations that deviated from the significant brain systems identified by forward and inverse models. Our findings offer crucial insights for applying machine learning to neuroimaging data, emphasizing the value of network enrichment for biological interpretation.

## INTRODUCTION

Machine learning (ML) statistical techniques offer great promise for predicting complex psychiatric and neurologic dysfunction using resting-state functional connectivity (rsFC) data (Arbabshirani et al., 2017; Bhaumik et al., 2017; Cropley et al., 2021; Guo et al., 2012). However, ML studies frequently report prediction accuracy, but fail to evaluate the biological validity or fundamental brain mechanisms leading to clinical and behavioral outcomes (He et al., 2020; Niu et al., 2020; Wang et al., 2013). While a growing number of studies are seeking to understand the brain regions that are most predictive of behavioral or clinical outcomes, biological interpretation of ML results remains a challenge for a myriad of reasons. Many neuroimaging studies have interpreted the relative importance of brain regions using the ML raw model weights (Bellantuono et al., 2021; Dhamala et al., 2020; Greene et al., 2020; Jiang et al., 2020; Kardan et al., 2022; Plitt et al., 2015). Though ML methods can identify the most predictive features, it has been shown that such interpretation could be seriously misleading from a biological perspective given that the raw ML weights may reflect nonneuronal or nuisance signal such as head motion or machine noise (Chen et al., 2022, 2023; Haufe et al., 2014; Siegel et al., 2017; Tian & Zalesky, 2021). In other words, raw weights from ML models only represent a combination of features for optimal prediction performance and should not be confused with the biological relation to the behavioral outcomes. Interpretable edge-level weights can either be derived from methods using traditional statistical models (i.e., forward models) or, for ML models, by calculating the covariance between the rsFC and the predicted outcome, hereafter referred to as an inversion model (Haufe et al., 2014). These methods provide feature interpretability for individual rsFC features. However, even with interpretable feature weights, one may be left with several thousand associations with behavioral or clinical outcomes and no readily available method to interpret the relative importance of certain brain regions relative to others.

At the rsFC systems level, analysis seeks to identify associations by leveraging the network-level architecture of the brain as described in several canonical atlases (Gordon et al., 2016; Power et al., 2011; Schaefer et al., 2018). These network atlases can be used to describe within-network and between-network connectivity, herein referred to as “network block” connectivity. An “individual network block” approach has been increasingly employed in the context of ML, in which only functional connections within a single network block (i.e., either within- or between-network rsFC) are used as features within the model (Kardan et al., 2022; Millar et al., 2020, 2022; Nielsen et al., 2019; Rudolph et al., 2018). Given that increasing the feature count can boost prediction accuracy, limiting ML models to only contain features within specific network blocks has resulted in limited accuracy (Bellantuono et al., 2021; Cui & Gong, 2018; Nielsen et al., 2019) and reliability (Mellema et al., 2021; Tian & Zalesky, 2021). In contrast, we present an innovative network-level analysis (NLA) method which uses all rsFC features in the connectome for ML modeling prior to performing statistical inference at the network level. NLA was recently developed for evaluating network-level enrichment in connectome-wide associations and has been successfully employed in several univariate association papers to understand connectome-wide associations with attention (Wheelock et al., 2021), emotion (Gilbert et al., 2021; Perino et al., 2021), gestational age (Wheelock et al., 2019), and autism (Eggebrecht et al., 2017; Marrus et al., 2018; McKinnon et al., 2019). However, the application of NLA to results produced from ML models remains unexplored.

To summarize, this paper proposes to combine ML with NLA to facilitate the biological interpretation of ML modeling in neuroscience data. We demonstrate the utility of the ML+NLA pipeline by modeling the association between the rsFC and age in Human Connectome Project (HCP) test-retest data taken from two scan days. We focused on age prediction in this study because age is the most reliable predictor with the largest effect size (Han et al., 2022; Liu et al., 2023; Monti et al., 2020) and has been investigated in numerous studies (Dosenbach et al., 2010; Smyser et al., 2016; Li et al., 2018; Lund et al., 2022). To ensure the effectiveness of this pipeline, we first evaluated the impact of feature filtering and sampling scheme on model performance. We then benchmarked network-level inference reliability using four edge-level statistical models: (1) simple Pearson correlations between rsFC and age, (2) linear support vector regression (LSVR) raw weights with *k*-best feature selection using Pearson correlations, (3) LSVR without feature selection, and (4) LSVR inverted weights. Finally, we compared NLA to the individual network block prediction model that models features from each network block independently and established the feasibility and utility of the novel combination of ML and NLA methods.

## MATERIALS AND METHODS

### Data Characteristics

The publicly available dataset from the HCP S1200 release was considered in the present study (Glasser et al., 2013). HCP study design included recruitment of twins and family members. Two 15-minute scans of resting-state fMRI were acquired on each of two separate days (here referred to as Rest 1 and Rest 2). This study design allowed for the assessment of test-retest reliability. A total of 965 healthy adults (ages 22–35 years old) were identified as having at least 10 minutes of low-motion data for both Rest 1 and Rest 2 of the HCP rs-fMRI data and were included for further analysis. Among all 965 participants, there were 420 families with a maximum of 5 members per family.

### Data Acquisition

High-resolution T1-weighted (MP-RAGE, 2.4 s TR, 0.7 × 0.7 × 0.7 mm voxels) and BOLD contrast-sensitive (gradient echo EPI, multiband factor 8, 0.72 s TR, 2 × 2 × 2 mm voxels) images were acquired from each participant using a custom Siemens SKYRA 3.0T MRI scanner and a custom 32-channel Head Matrix Coil. The HCP employed sequences with both left-to-right and right-to-left phase encoding, with each participant completing a single run in each direction on two consecutive days, which resulted in a total of four runs including two runs for Rest 1 and another two for Rest 2 (Van Essen et al., 2012).

### Data Processing

Minimally preprocessed data have been shown to be insufficient in controlling for confounds such as subject head motion (Burgess et al., 2016). Additional research suggests that sufficient low-motion functional connectivity data must be available for each subject in order to make reliable claims about associations between functional connectivity and behavior or outcomes (Gordon et al., 2017; Laumann et al., 2015). In this section, we provide the preprocessing steps of the dataset.

#### Functional connectivity preprocessing.

The functional MRI data preprocessing methods employed in this study have been previously described (Seitzman et al., 2020). First, to account for magnetization equilibrium and any responses evoked by the scan start (Laumann et al., 2015), the first 29.52 seconds, or 41 frames, of each resting-state run were discarded. Then, the functional data were aligned to the first frame of the first run using rigid body transforms, motion corrected (3D cross-realigned), and whole-brain mode 1,000 normalized (Miezin et al., 2000). The data, consisting of 2 × 2 × 2 mm voxels, was then registered to the T1-weighted image and a WashU MNI atlas using affine and FSL transforms (Smith et al., 2004).

Further preprocessing of the resting-state BOLD data was applied to remove artifacts (Ciric et al., 2017; Power et al., 2014). Specifically, frame-wise displacement (FD), a metric used to quantify the amount of motion or displacement between consecutive frames in fMRI data, was calculated (Power et al., 2012), and artifact removal (Ciric et al., 2017; Power et al., 2014) was completed with a low-pass filter at 0.1 Hz to address respiration artifacts affecting the FD estimates (Fair et al., 2020; Siegel et al., 2017), along with a threshold after the low-pass respiration filter to remove frames with FD greater than 0.04 mm (Dworetsky et al., 2021). To prepare the data for functional connectivity (FC) analysis, the regression of nuisance variables was performed, including 36 regressors: (1) three time series (whole-brain mean, mean ventricular CSF, mean white matter) with temporal derivatives from the Volterra expansion (12 total parameters), and (2) six head motion parameters with temporal derivatives from the Volterra expansion (24 total parameters) (Friston et al., 1996; Satterthwaite et al., 2012; Yan et al., 2013). Spatial masks of the gray matter, white matter, and ventricles were created from the T1-weighted images for each of the individual-specific regressors using Freesurfer 5.3 automatic segmentation (Fischl et al., 2002). Segments of data lasting fewer than five contiguous frames were excluded, and then least squares spectral estimation was used to interpolate over the censored frames (Hocke & Kämpfer, 2008; Power et al., 2014). Data were then band-pass filtered from 0.009 to 0.08 Hz, and censored frames were removed from the time series (Seitzman et al., 2020). We emphasize that if censoring and interpolation were not performed prior to filtering, the filter would smear high-motion noise artifacts into adjoining frames. To correct this issue, one would need to censor high-motion frames as well as three to five frames on either side of each high-motion time point. Censoring and interpolating prior to filtering can effectively prevent this problem.

Following previously established methods (Gordon et al., 2016), the preprocessed BOLD time series data underwent surface processing, which involved using the ribbon-constrained sampling procedure in Connectome Workbench to sample the BOLD volumes to each subject’s individual native surface and exclude voxels with a time series coefficient with a variation 0.5 *SD*s above that of the mean of nearby voxels (Glasser et al., 2013; Gordon et al., 2016). After being sampled to the surface, time courses were then deformed, resampled, and smoothed using a Gaussian smoothing kernel (FWHM = 4 mm, sigma = 1.7). Connectome Workbench was then used to combine these surfaces with volumetric subcortical and cerebellar data into the CIFTI format to create full brain time courses, excluding non–gray matter tissue (Glasser et al., 2013).

#### Whole-brain rsFC feature extraction.

**Figure 1.**
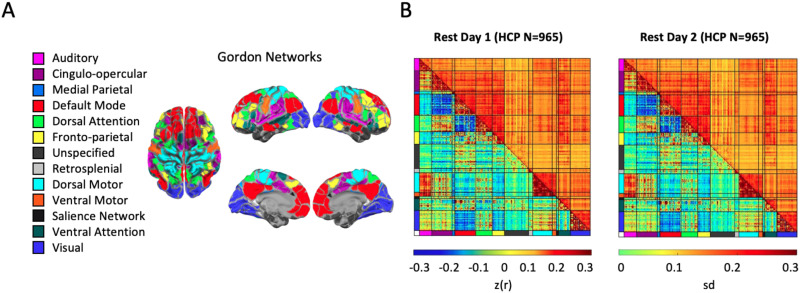
Resting-state functional connectivity data. (A) Gordon networks. As defined in Gordon et al. (2016), 333 parcels in the brain were used to extract mean rsFC and were grouped into 12 networks and 1 unspecified network. (B) Mean and standard deviation for rsFC on two separate days. For each pair of 333 parcels, the mean (lower triangle) and the standard deviation (upper triangle) for the 10 minutes of low-motion data rsFC data were computed (the Pearson correlations were standardized by Fisher’s *z* transformation and denoted by *z*(*r*)) across all 965 HCP participants on two different scan days.

After the processing procedure above, surface-based parcels and canonical functional networks (Gordon et al., 2016) were used to parcellate a set of 333 previously defined regions of interest (ROIs) into 12 networks and 1 unspecified network as shown in Figure 1A, where the unspecified network consists of unassigned parcels that were not strongly connected with any other parcels as defined in Gordon et al. (2016). For each subject, the mean time series within each ROI was calculated by taking the average of time series over all the voxels within this ROI. Since we randomly sampled 10 minutes of low-motion frames from each ROI for each subject on two scan days, all 965 participants had the same amount of low-motion data on both days. The Pearson correlation between the mean time courses of each pair of ROIs was evaluated (55,278 pairs in total, ROIs on the diagonal excluded) and normalized with a Fisher z-transformation. A correlation matrix consisting of these normalized correlations was constructed for each of the two scan days, respectively, across all the 965 participants. For each scan day, the average and standard deviation of all 965 correlation matrices are shown in Figure 1B.

### Machine Learning Model

#### Linear support vector regression.

Rather than fitting a linear regression model on each feature independently, we instead utilized a linear support vector regression (LSVR) to model the relationship between multiple features (i.e., an individual’s rsFC) and chronological age. Support vector regression (SVR) is an extension to support vector machine, which is frequently applied to classification problems with binary labels, such as testing if an individual can be identified as a part of a specific category based on the rsFC. Since the predicted response in our case (i.e., chronological age) is a continuous variable, we employed SVR to fit an ML regression model and predict the continuous value. Various kernel functions can be chosen when applying SVR, such as linear kernels, sigmoid kernels and Gaussian kernels (RBF). Among them, linear SVR is commonly employed in rsFC studies since it is less prone to overfitting than nonlinear ones and is significantly faster to train (Cui & Gong, 2018; Taxali et al., 2021; Wang et al., 2013).

For all approaches detailed below, we divided the data using an 80% training and 20% test holdout procedure and repeated this 1,000 times. The hyper-parameter in the LSVR model (i.e., the tuning parameter of the L^2^ Ridge penalty term) was selected by a fivefold cross-validation within the training data on each repetition. Specifically, on each repetition within the training set containing 80% of the participants, we further randomly divided the data into an inner cross-validation training set with 80% participants and an inner-cross-validation test set with 20% participants. We tried different hyper-parameters in a certain range to fit the LSVR model using the inner-training set and picked the optimal one with the minimized mean square error (MSE) yielded by the prediction using inner-test set for each repetition. We refer to Figure S1 in the Supporting Information for the clarification of this nested fivefold cross-validation method. To evaluate the performance of LSVR models, we adopted four different measures: (1) prediction accuracy by the Pearson correlation between the predicted and actual ages, (2) prediction accuracy by the mean absolute error (MAE), (3) variance of the prediction by MSE, and (4) reliability score of brain-age predictions, that is the intraclass correlation statistic (ICC). Specifically, we choose the type ICC(2, 1) in this study because we are predicting an outcome, and our goal is to evaluate the reliability of brain-age predictions instead of the reliability of rsFC (Koo & Li, 2016; Taxali et al., 2021). For each repetition, we first fit the ML models using the training set and then calculated the evaluation measures above in the test set by comparing the predicted age and the actual age.

#### Sampling scheme for cross-validation and training/testing.

A considerable number of large-scale neuroscience studies are designed to leverage twins and families with heritable characteristics. For example, twins and siblings are present in cohorts such as the Infant Brain Imaging Study (Eggebrecht et al., 2017), Adolescent Brain Cognitive Development (ABCD) study (Hahn et al., 2022), and HCP (Cohen et al., 2020; Feilong et al., 2021; Greene et al., 2020; Van Essen et al., 2012). Such shared variance due to families or longitudinal designs (repeated measures within a subject) poses a challenge to ML modeling, especially for out-of-sample testing such as cross-validation (CV) and random sampling. Neuroscience research has inconsistently employed methods to address this shared variance within ML models. For instance, some studies do not account for the dependency of related individuals when implementing ML models (Cui & Gong, 2018; Scheinost et al., 2019), which violates the fundamental assumption of that the train and test sets should be independent. This oversight can lead to inaccurate model estimation and inflate prediction accuracy. Alternatively, some studies have reduced the total sample size by only including nonrelated individuals, such as retaining only one family member (Dhamala et al., 2020; Elliott et al., 2019; Gbadeyan et al., 2022; Li et al., 2017; Lohmann et al., 2021; Nostro et al., 2018; Seitzman et al., 2020; Tian & Zalesky, 2021). This approach may reduce prediction power due to decreased sample size, defeats the purpose of twin study designs, and, in the presence of high-dimensional data, unnecessarily halving datasets can worsen the overfitting issue, triggering bias and unreliability (Marek et al., 2022). Finally, some studies have considered shared variance in ML models. One method is to leave-one-family-out, which resembles the traditional leave-one-out CV, but incorporates controls for family structure (Dubois et al., 2018; Feilong et al., 2021; He et al., 2020). However, previous studies (e.g., Varoquaux et al., 2017) have suggested that the leave-one-out CV can lead to large variance between repetitions due to the high correlation among training sets (training sets would only differ by one data point). A second method to modeling shared variance keeps family members together in either the CV folds or training or test set but does not allow family members to be in both (O’Connor et al., 2021). Various approaches such as the grouped K-fold CV, stratified grouped K-fold CV, and leave-one-group-out CV, are readily available (e.g., as included in the Python package “scikit-learn”) and aim to repeatedly shuffle and split the data into grouped train and test partitions. However, while methods are available to account for dependency, these methods are not uniformly employed.

**Figure 2.**
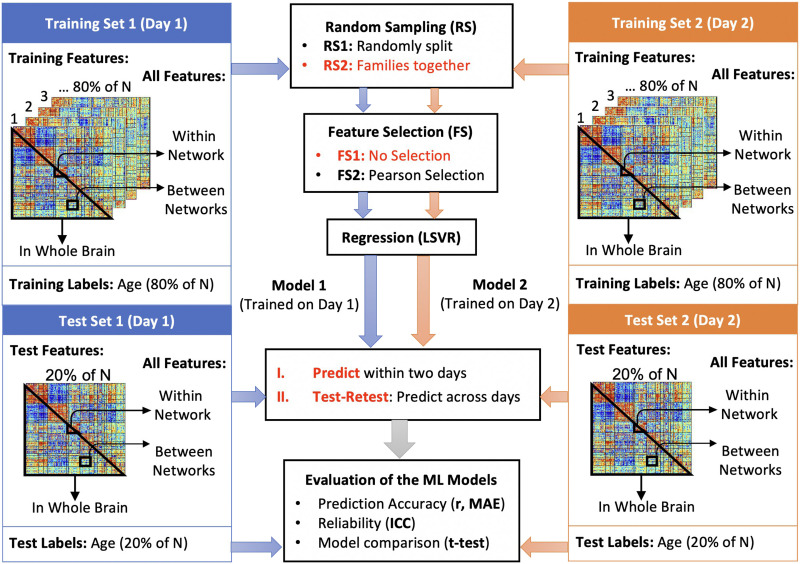
Overview of the machine learning (ML) pipeline. Linear support vector regression (LSVR) was employed to fit a regression model for the relationship between rsFC matrices and the ages on two separate scan days (Rest day 1 and Rest day 2). On each day, we randomly sampled 80% of the participants, assigning them to a training set, and the remaining 20% participants were assigned to a test set. The subsampling on two scan days were independent of each other. Two different random sampling (RS) mechanisms were applied for comparison. One is to randomly split the participants to the training and test sets without considering the family structure (RS1), and another one is to keep family members together within either training or test set (RS2). Both RS procedures were repeated for 1,000 repetitions, respectively. Within the training set of each repetition, the hyper-parameter was chosen by an embedded fivefold cross-validation (CV). For the test-retest, we trained an LSVR model on Rest 1, predicted the ages on Rest 2, and fit another LSVR model via the training set from Rest 2 and tested on Rest 1, respectively. We averaged the prediction accuracy of two predicted sets to yield the ICC(2, 1). The corrected resampled *t* tests were performed to compare the differences between the resampling-based ML models.

To test the impact of shared variance across family members on the ML model performance, we employed two different sampling approaches. In the first sampling approach, we assigned participants to the training or test set randomly ignoring family structure, an approach that is commonly used across datasets including HCP (Cohen et al., 2020; Cui & Gong, 2018). We performed the random assignment for 1,000 repetitions. In the second sampling approach, we used an identical 80%–20% training-test split, but assigned families to either the training or test set, not both (i.e., family members were never in both the training and testing sets) in order to account for the family structure within the HCP dataset (Figure 2). The sampling procedure was also performed with 1,000 repetitions (Supporting Information Figure S1).

#### Feature selection.

A second challenge arises from the vast number of features typically found in rsFC across the entire brain, amounting to several thousand, necessitating the need to address issues such as overfitting and excessive computational requirements in many ML models. Consequently, a feature selection step is frequently implemented prior to the ML model in order to reduce the number of rsFC features included in the regression model (Arbabshirani et al., 2017; Craddock et al., 2009; Gao et al., 2019). Many feature selection methods fall into the category of massively univariate approaches that model the relationship between each rsFC feature and predictive outcome independently, such as marginal Pearson screening with the top-ranked features selected for inclusion in the ML model (Fan et al., 2006; Nielsen et al., 2019; Shen et al., 2017). However, a notable concern of such feature selection methods is that they may exclude features that have a significant contribution in a full high-dimensional regression model, particularly when the predictors exhibit high correlation (Wang et al., 2021).

In order to understand the impact of feature selection on prediction accuracy, reliability, and biological interpretability, we employed two approaches (Figure 2). In the first approach, we employed the *k*-best feature selection with Pearson correlation on the training set in which we ranked features according to the marginal Pearson correlation between each functional connection and the age for all participants in the training set. Then, we selected the *k* = 1,000 top ranking features (strongest univariate correlations between rsFC and age) to enter the LSVR model since the performance of an LSVR model has been shown to converge when the number of predictors surpassed 1,000 (Cui & Gong, 2018; Nielsen et al., 2019). For brevity, we shall call this method “Pearson feature selection” in the rest of the paper. We continued to fit the LSVR model by the same training set that we used for feature selection to ensure that, in each of the 1,000 repetitions, the test data was never touched before prediction. In the second approach, we applied no feature selection and allowed all rsFC features within the lower triangle of the rsFC matrix, excluding the diagonal, to enter into the LSVR model (55,278 features).

Furthermore, given that Pearson correlation is also frequently used as a univariate method to investigate the relationship between rsFC and the predictive outcomes (i.e., age), we further compared the significant features selected by the univariate correlation approach (i.e., Pearson) and the multivariate regression model (i.e., LSVR) with and without Pearson feature selection.

#### Interpretation of ML model weights.

In contrast to methods such as Pearson correlation or ordinary least squares regression, which fall in the category of forward models in the multivariable data analysis (Haufe et al., 2014), it can be severely misleading to provide biological interpretations for the raw ML model weights (Jiang et al., 2020; Sripada et al., 2019) or selected features (Finn et al., 2015; Greene et al., 2018) of the backward multivariable ML models. Roughly speaking, forward models investigate how the observed variables are driven by the underlying factors, while backward models focus on expressing the variables of interest as a function of the observed data. Therefore, the interpretability of an ML model is determined by the direction of the functional relationship between observations and underlying variables: the weights of forward models are interpretable, while those of backward models are not in most cases. Due to this distinction, an inversion process is necessary to facilitate reasonable interpretations for the backward models. We followed the Haufe’s inversion model (Haufe et al., 2014) to resolve this interpretational issue. Specifically, we transformed our backward LSVR model into the forward form by computing the covariance between the predicted target variable age and the rsFC (Chen et al., 2022). We point out that the rsFC-age covariance by the inversion model is different from the univariate Pearson correlation model, since the latter is computed between each rsFC and actual age, while the former is between each rsFC and the predicted age estimated from the LSVR model, which included all rsFC from the connectome.

#### Test-retest reliability.

We assessed the reliability of our ML model by evaluating its performance consistency across different participants and days. Given the samples from two scan days, we first conducted the sampling and cross-validation on two days separately to obtain two training sets and two test sets. We took the average of the predicted test set on Rest 1 using the trained model from Rest 2 and the predicted ages from the test set on Rest 1 using the trained model from Rest 2 (Figure 2). Since we proposed to compare two different sampling implementations and two LSVR models with or without feature selection, we would expect in total 12 sets of predicted outcomes for all participants (two sampling approaches, two feature selection approaches, and two scan days plus one test-retest between scan days).

ICC score is another standard metric to assess reliability, and among various forms of ICC scores, ICC(2, 1) is utilized in our study to evaluate the consistency of predicted outcomes from LSVR models for all participants across two scan days, which is based on the guidelines for ICC scores in previous studies (Koo & Li, 2016; Taxali et al., 2021). To obtain ICC(2, 1) scores, we first predicted the values in the test set on Rest 2 via the LSVR model trained on Rest 1, repeated the same procedures on the training set from Rest 2 and the test set from Rest 1, and then calculated the ICC(2, 1) statistic according to the rigorous formula available in the Supporting Information.

In order to understand the impact of statistical model choice (univariate, multivariable prediction with feature selection, multivariable prediction without inversion, multivariable prediction with inversion) on the spatial patterns of rsFC associations with age, we plotted the results from our statistical models spatially on the connectome. Specifically, for each model, we binarized all the rsFC features (“1” if the absolute value of *Z*-score larger than 2, i.e., |Z| > 2, “0” otherwise). We repeat this binarization for all 1,000 repetitions of the Rest 1 data and 1,000 repetitions of the Rest 2 data. Then, for each rsFC feature, there is a binary vector of length 2,000 to indicate if it is significant in all 2,000 repetitions. We counted the nonzero elements of this binary vector and divided it by 2,000, which results in the overlapped percentage for each rsFC feature. We then repeat the same procedure for all the rsFC features to form the overlapped percentage matrix.

#### Multiple comparison of resampling-based ML models.

To evaluate the different ML models implemented by subsampling techniques, we adopted the corrected resampling *t* test (Nadeau & Bengio, 2003) to examine if there is significant difference between the ML models. The Student’s *t* test is not valid because the training and test sets subsampled from the original data can overlap in different repetitions, and hence they are not independent. This violates the independence assumption required in the classic Student’s *t* test, which may result in underestimation of the variance of differences, and Type I error can exceed the significance level. To avoid this issue, we corrected the variance estimate by taking the dependency between subsamples into account (Nadeau & Bengio, 2003) and calculated the corrected resampling *p* values along with the Bonferroni correction as the measure of significance. We refer to the Supporting Information for the details of this corrected test. To deal with the multiple tests, we divided the significance level *α* by 6 (the number of tests we were performing to compare four different models) as the Bonferroni-corrected significance level *α*_mod_ = 0.00833.

### Network-Level Enrichment Analysis

Network-level analysis (NLA) employs enrichment techniques to assess if pairs of networks exhibit significant clustering of robust brain-age (or other behavior) correlations. This approach utilizes standard statistical tests to evaluate the concentration of associations within specific network pairs, such as the chi-square (*χ*^2^) test and Hypergeometric test, thereby focusing the analysis on fewer, more relevant comparisons at the network level. We refer to more details of the NLA methodology in the Supporting Information. NLA has been previously used to determine brain networks associated with behavioral outcomes by modeling univariate rsFC correlations (Eggebrecht et al., 2017; Marrus et al., 2018; McKinnon et al., 2019; Wheelock et al., 2018, 2019, 2021, 2023). In a novel application, to facilitate accessible biological interpretation of the estimated weights of rsFC features, we applied NLA toolbox to the results from our ML models, which used the features across the entire connectome.

#### Observed network-level enrichment.

Using the methods described above, we generated four sets of inputs for NLA to evaluate the significant network blocks predictive of age. These methods included (1) Pearson correlation, (2) Pearson correlation feature selection + LSVR, (3) LSVR without feature selection, and (4) LSVR with inversion. Specifically, for the first method, we calculated the Pearson correlation between each rsFC feature and the age. For the second method, we applied the Pearson feature selection ahead of LSVR model fitting as described above in the section Feature Selection. Then we trained the LSVR model and estimated the weights of the features that were included in the model (i.e., the features that have been selected in the screening step). For the third approach, we estimated the weights of all 55,278 features by the LSVR model without feature selection. For the last approach, we used the predicted age from the LSVR model without feature selection, and computed the covariance between each rsFC edge and the predicted age in order to estimate the inverted feature weight corresponding to each rsFC edge (Haufe et al., 2014). For the Pearson correlation model, we calculated the mean FC-age correlations across 1,000 resampling repetitions as the input while for the other three models we used the mean estimated FC-age weights across 1,000 repetitions to be the input.

**Figure 3.**
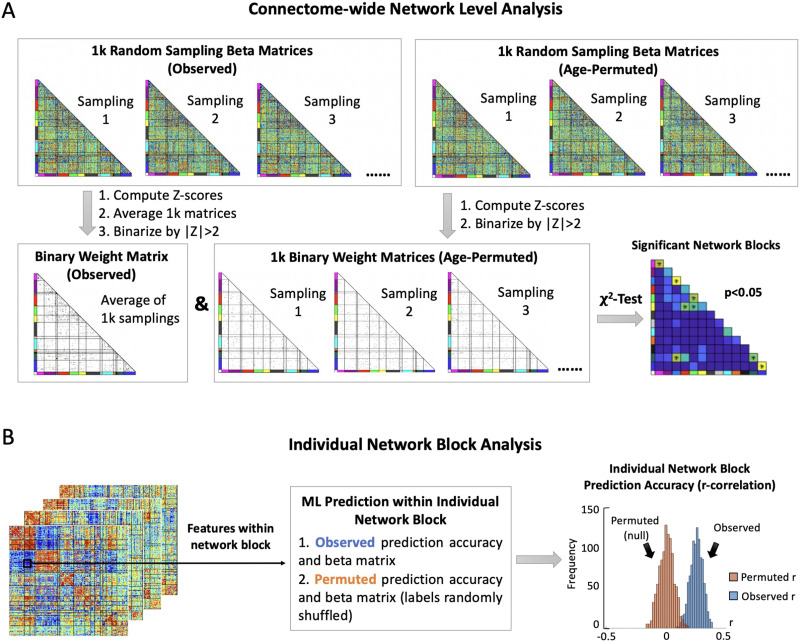
Localizing predictions to biological systems. For the purposes of determining significant network blocks toward prediction and biological interpretation, two different network block analysis methods were employed as comparison. (A) Connectome-wide network-level analysis (NLA). The NLA software used the output from the method illustrated in Figure 2 which was repeated 1,000 times with response outcomes (age) randomly shuffled to generate permuted weights. Both observed and permuted weights were *Z*-scored and binarized at a threshold of |Z| > 2. These thresholded and binarized *Z*-scores were then used as inputs to a *χ*^2^ test, yielding a *χ*^2^ test statistic and a *p* value. Then, a permutation-based FWER control was performed, and the network blocks with this FWER-controlled *p* value < 0.05 were considered as significantly predictive of age. (B) Individual network block analysis. In contrast, for each individual network block, we fit a ML model with the features from within this certain network block by applying the same random sampling and cross-validation implementation as the ML pipeline in Figure 2. We then compared this observed distribution of raw ML model weights to a permuted null distribution of raw model weights generated by fitting a ML model with features only from this network block but on randomized ages. We evaluated the prediction accuracy of each network block prediction model with the prediction accuracy quantified by correlating the observed and predicted ages. The same procedures were performed another 1,000 times for each model to obtain permuted *r* correlations.

Given the weights of rsFC features as input of NLA, we next introduce the detailed NLA procedure for analysis. These weights were first *Z*-scored, thresholded at |Z| > 2 and then binarized (Figure 3A). We selected the threshold value of 2 after experimenting with several thresholds. Among them, 2 provided a balanced distribution of features throughout the connectome, striking a suitable balance between sparsity and density that qualitatively resembled the sparsity in previous univariate correlation analysis studies using a significance level of *p* < 0.05 (Eggebrecht et al., 2017; Wheelock et al., 2021, 2023). We also examined the effect of different *Z*-score thresholds in Figure S2. Weights of rsFC features within each network block passing this threshold were used to compute the *χ*^2^ value relative to the distribution of all weights passing the threshold in the rest of the connectome. The 1-degree-of-freedom *χ*^2^ test was used to compare the observed number of nominally strong (thresholded and binarized) weights of rsFC features within a pair of functional networks to the number that would be expected if rsFC features with strong weights were uniformly distributed across the full connectome (Eggebrecht et al., 2017; Wheelock et al., 2021). A large resulting test statistic can indicate that the number of strong associations within a specific network block is enriched, meaning the number of nominally strong weights is much greater than expected.

#### Permutation test.

To evaluate the network-level significance of each network block, we adopted the permutation test to compute the permutation-based family-wise error rate (FWER) controlled *p* values. To generate the permuted data, we shuffled the ages and fit the same model (Pearson correlation, LSVR no feature selection, LSVR with Pearson feature selection, or inverse LSVR) with the whole-connectome data to create null brain-age weight matrices. This procedure was repeated for 1,000 repetitions for each model, respectively. The *χ*^2^ test statistics were also calculated on permuted models, generating a null distribution of network-level statistics. The observed (i.e., real) *χ*^2^ values were then compared to the null distribution to establish network-level significance for *p* < 0.05. Additional details of this FWER-controlled *p* values are described in the Supporting Information.

Furthermore, we quantified network-level reliability using Matthews correlation coefficient (MCC). We first determined the confusion matrix by treating Rest 1 as the observation set and Rest 2 as the prediction set. Accordingly, the significant blocks in Rest 1 were denoted as “true” and other blocks as “false.” Then, significant blocks in Rest 2 were denoted as “positive” and other blocks as “negative.” Specifically, the blocks with *p* < 0.05 in Rest 1 and Rest 2 were designated “true positive” (TP), and the ones with *p* ≥ 0.05 in Rest 1 and Rest 2 were “true negative” (TN). Then, the blocks with *p* < 0.05 in Rest 1 while with *p* ≥ 0.05 in Rest 2 were “false negative” (FN), and the ones with *p* ≥ 0.05 in Rest 1 while with *p* < 0.05 in Rest 2 were “false positive” (FP). With this configuration, we can calculate MCC scores by the formula MCC = (TP * TN − FP * FN)/$\sqrt{\left(\text{TP}+\text{FP}\right)\left(\text{TP}+\text{FN}\right)\left(\text{TN}+\text{FP}\right)\left(\text{TN}+\text{FN}\right)}$. The MCC ranges from 0 to 1 with 1 being more consistent across two scan days.

It is worth noting that many investigators have studied the proper ways to draw a null distribution. Xia et al. (2018), for example, shuffled the networks in addition to permuting the predictive outcomes to generate the null distribution; Zamani Esfahlani et al. (2020) summarized different ways to create a null model that could preserve neuroanatomical realism; and more recently, Váša et al. (2021) provided a comprehensive review on the logic, implementation, and interpretation of null models for functional connectomes. Here, we have not shuffled the functional networks of the brain in order to preserve the underlying covariance structure of real biological data (Nichols & Hayasaka, 2003). We examined the impact of shuffling FC networks for the permutation test as in Xia et al. (2018) and observed that the resulting permuted data did not resemble the covariance structure of real data (Figure S3).

#### Network block feature analysis.

In contrast to NLA which uses connectome-wide model weights as input, prior work seeking to localize important features to biological systems has used only the features within an individual network block, with an emphasize on network block prediction accuracy (Millar et al., 2020, 2022; Nielsen et al., 2019; Rudolph et al., 2018). An “individual network block” test uses the rsFC within a single network block to generate prediction *r correlations* between predicted and true labels. These observed network block results are then compared to a null distribution generated from rsFC in the same network block with outcome labels permuted. If a significant shift is observed between these two *r distributions*, then it can be inferred that this specific network block contributes significantly to the prediction of response outcomes (i.e., ages) (Figure 3B). Specifically, an individual network block analysis that was performed on the within- and between-network rsFC features of 13 functional networks would fit 78 individual predictive models (i.e., a model for a network block), and these 78 prediction accuracies would be compared to 78 corresponding null distributions to determine significant predictive network blocks. In contrast, NLA conducts statistical inference tests on network blocks after a connectome-wide model has been fit using all rsFC as features. Furthermore, NLA compares the weights in each network block to a null model generated from connectome-wide permutation testing to establish significance. For brevity, we call the former an individual network block analysis and the latter NLA method a “connectome-wide” analysis.

To show that prediction-oriented individual network block methods may be invalid, we performed a further analysis on the network blocks that exhibit predictive significance compared to the null distribution using permuted predictive outcomes. We adopted a more stringent evaluation to determine the significance of a network block by comparing it to randomly selected features outside of this certain network block. Given that prediction accuracy is a function of feature set size (Domingos, 2012; Guyon & Elisseeff, 2003; Nielsen et al., 2019), we compare it to an equally large feature set selected from the rest of the connectome external to the network block being tested. In particular, we first conducted the individual network block analysis, and for each significant block yielded by this approach, we further generated a different null model that randomly selected the same number of features from the full connectome excluding all the significant blocks. We compared the distribution of prediction accuracy from the network block and a set of randomly selected features of the same count. If the random model outperforms the observed one, then we can conclude that the individual network block type methods might not be valid for biological inference.

## RESULTS

### Shared Variance Among Families and Use of Feature Selection Impacted Prediction Performance

**Figure 4.**
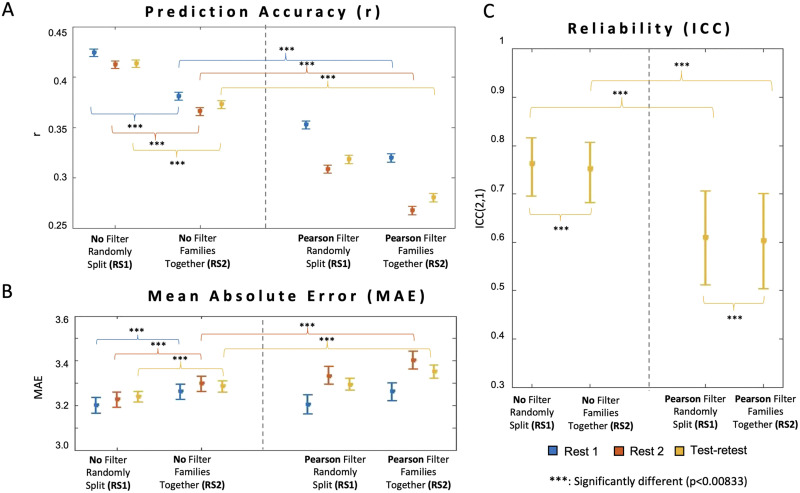
Evaluations of the ML models with two sampling strategies and two feature selection approaches. (A) Pearson *r* correlation between the actual and predicted outcomes, with error bars indicating the standard deviation of r correlations. (B) The mean absolute error (MAE) by the model. (C) The reliability of the model quantified by ICC(2, 1) for two different days, with error bars indicating the standard deviation of ICC(2, 1) scores. The corrected resampled *t* tests were performed to compare different models across conditions (six total tests), with the significance result of 95% confidence level between the models indicated by *** if the corrected *p* value is smaller than 0.0083.

When comparing the model performances with the random sampling schemes considering the family structure (RS2) and not considering the family structures (RS1), we observed that training the ML model without taking the shared variance among related participants into account led to falsely inflated prediction accuracy (*r*, MAE) and reliability (ICC) (Figure 4A–C; Supporting Information Tables S1 and S2). The mean correlation values (over 1,000 repetitions) for the ML models using RS1 are 0.4147, 0.3968, and 0.4019 for Rest Day 1, Rest Day 2, and test-retest respectively, while the ones using RS2 are 0.3755, 0.3552, and 0.3669 (no feature selection). Furthermore, when a marginal Pearson feature selection was applied ahead of LSVR fitting, we noticed a significant decrease (*p* value from the corrected resampled *t* test < 0.0083) in the prediction accuracy (*r*) and reliability (ICC) (Figure 4A–C). Specifically, the mean correlation values for the ML models with Pearson feature selection are only 0.3113, 0.2430, and 0.2654 (RS2). Similar decreases were observed using RS1.

### Locations of Strong Edge-Level Model Weights Differed According the Estimation Method

**Figure 5.**
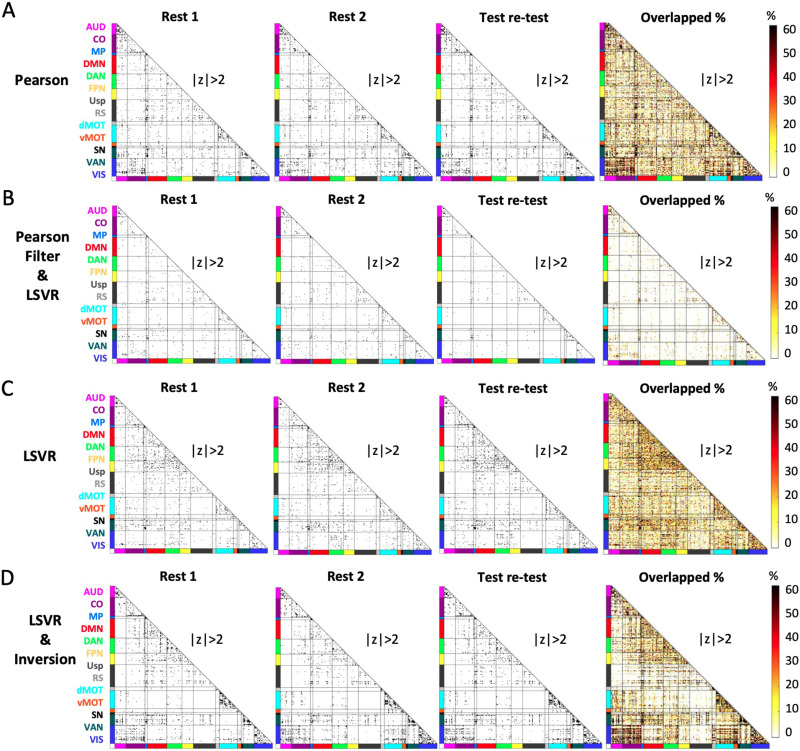
Nominally significant rsFC features selected by four different methods on two days. (A) Pearson *r* correlation. (B) Pearson *r* correlation feature selection and LSVR. (C) LSVR with no feature selection. (D) Inversion of LSVR model weights without feature selection. All results were *Z*-scored, taken absolute values, and thresholded at |Z| > 2 for standardized analysis and display of rsFC-age associations across different methods. Test-retest was performed by the scheme in Figure 2. Overlapped % of each rsFC feature over all the random sampling repetitions and two scan days were calculated and visualized as heat maps in the last columns of panels A, B, C, and D. Details of the overlapped % calculation can be found in the Supporting Information.

We investigated the associations of rsFC features to the predictive outcome (age) via four different measures, including (1) the univariate marginal Pearson correlation between each rsFC feature and age, (2) using the top 1,000 ranked univariate Pearson correlations as a feature selection step for LSVR, (3) all rsFC entered as features predicting age, and (4) all rsFC entered as features predicting age with inversion of the LSVR model. Patterns of strong univariate marginal Pearson correlations between rsFC and age qualitatively appeared to cluster within network blocks along the diagonal and in off-diagonal network blocks including visual-auditory (VIS-AUD) and visual-cingulo-opercular (VIS-CO) (Figure 5A). As expected, we observed that the distribution of the rsFC features with the strongest weights obtained by “Pearson feature selection + LSVR” appeared in the network blocks similar to those where the strong univariate Pearson correlations appeared. By definition, given that we only selected the 1,000-strongest Pearson correlations for feature selection, the results of Pearson feature selection + LSVR were sparser than the other models (Figure 5B). In contrast, the LSVR model on the full connectome (without feature selection) yielded a very different pattern of results relative to the other three models. Specifically, rsFC features with strong weights tended to be clustered in network blocks such as the default mode network (DMN), dorsal attention network (DAN), and fronto-parietal network (FPN) (Figure 4C). This showed that a prestep of feature selection before applying the LSVR model could dominate the estimation results from the ML algorithm. However, strong raw ML model weights cannot be interpreted as corresponding to neural predictors of age (Chen et al., 2022; Haufe et al., 2014). Therefore, we applied the inversion to the LSVR model to obtain the multivariable weights in which the directionality and magnitude can be interpreted. We observed that the spatial pattern of rsFC features most predictive of age after inversion (Figure 4D) were in similar network blocks to those from the univariate marginal Pearson correlation (Figure 4A) and the raw LSVR weights (Figure 4C).

### The Inherent Covariance Structure of rsFC Data Should Not Be Removed During the Permutation Test

For the univariate Pearson correlation, the LSVR models with and without inversion applied, we visually inspected our permuted weight matrices prior to analysis. We observed that randomization of the ages resulted in permuted Pearson correlations results that were relatively stronger in some network blocks than others, but that the network blocks with these strong associations changed across permutations. In contrast, we observed consistent patterns of strong estimated LSVR weights and inversed LSVR results within consistent network blocks across permutations even with the predictive outcomes (ages) randomly shuffled (Figure S3). However, when we randomized the ages and shuffled the networks in the connectome using the method described in Xia et al. (2018) the resulting permuted connectivity matrices for both Pearson and LSVR models exhibited a relatively uniform distribution across networks and did not retain the underlying covariance structure of the data. Given the lack of biological realism of these weight matrices, the method of randomly shuffling networks was not used for permutation testing in subsequent analyses.

### Network-Level Enrichment Characterized Networks Most Predictive of Age Across Models

**Figure 6.**
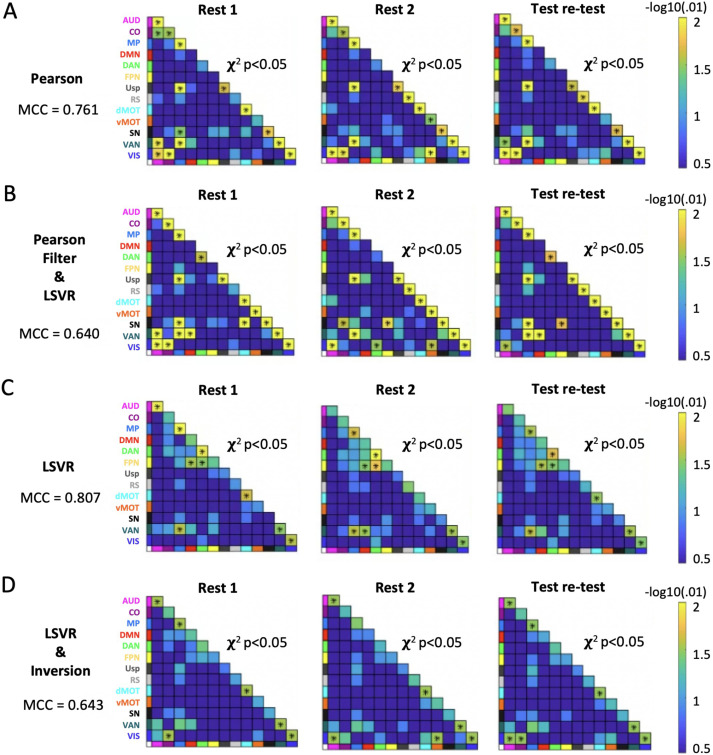
Significant network blocks selected on two scan days by network-level analysis (NLA) with four different sets of inputs. Specifically, the inputs were obtained from (A) Pearson *r* correlation, (B) linear support vector regression (LSVR) model with Pearson feature selection applied ahead, (C) LSVR model, and (D) LSVR model with inversion. The Mathews correlation coefficients (MCC) were also calculated for each method.

**Figure 7.**
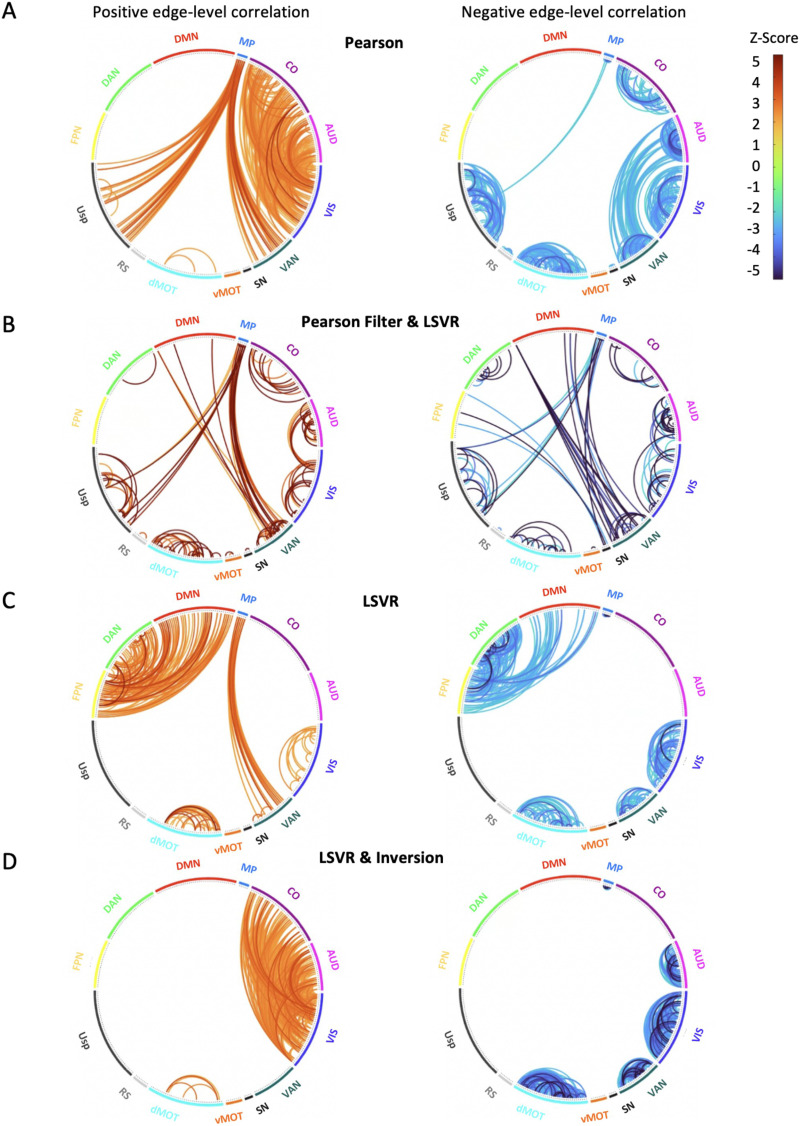
Edge-level chord plots of the significant network blocks selected by the four models. Positive (left) and negative (right) edge-level correlation (*p* < 0.05) are shown, respectively, within the significant network blocks by the following four models: (A) Pearson *r* correlation, (B) LSVR model with Pearson feature selection applied ahead, (C) LSVR model, and (D) LSVR model with inversion. All the chord plots correspond to the test-retest column in Figure 6.

NLA with four different sets of inputs (i.e., Pearson, Pearson feature selection + LSVR, LSVR, and inverted LSVR) characterized different network blocks that were most predictive of the age. Pearson correlations and the weights of rsFC features from the LSVR model with Pearson feature selection resulted in similar significant network blocks, consisting largely of within-network blocks on the diagonals (Figure 6A and B). In contrast, the NLA with weights from LSVR model as input selected distinctly different networks, such as DAN, FPN-DMN, and FPN-DAN (Figure 6C). When applying the inversion technique to the LSVR model for biological interpretation (Chen et al., 2022; Haufe et al., 2014), we observed that it yielded several significant network blocks that were consistent with the marginal Pearson and Pearson feature selection + LSVR methods and inconsistent with the spatial pattern of network-level results from LSVR without inversion, including auditory-visual (AUD-VIS) and cingulo-opercular-visual (CO-VIS) (Figure 6D). For each of the four methods, we assessed the network-level reliability by the MCC scores, which were 0.761, 0.640, 0.807, and 0.643, respectively, as shown in Figure 6. The chord plots of the significant network blocks tested by NLA with these four different sets of outputs are presented in Figure 7.

### Interpretations of Significant Network Blocks Cannot Rely on the Individual Network Block ML Models

**Figure 8.**
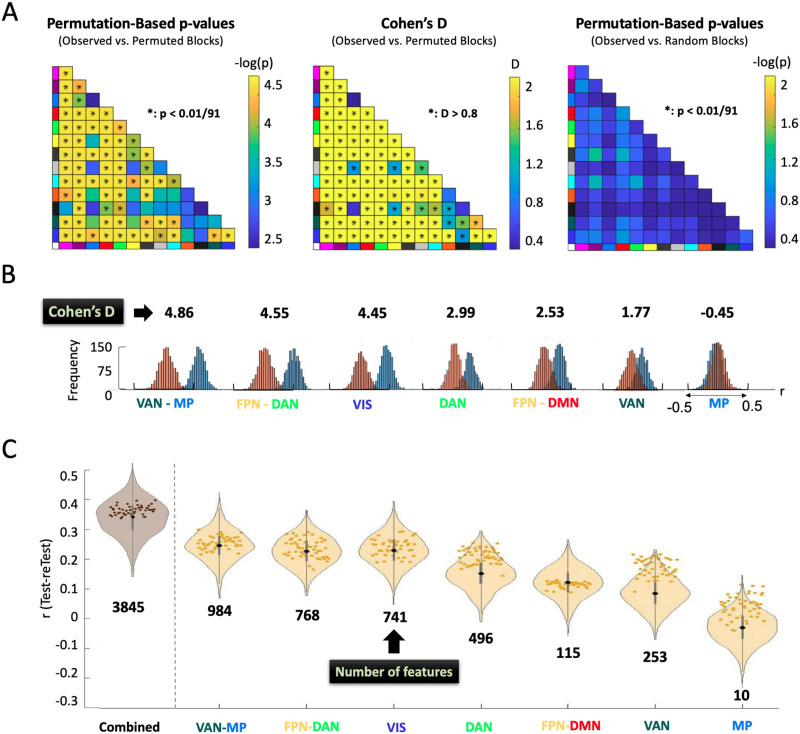
Results of the individual network block analysis. (A) Results of three different hypothesis tests from the “individual network block” analysis. The Permutation-based *p* values and Cohen’s *d* values were computed with reference to the null distribution from permuting the ages. The last plot computed the FWER-controlled *p* values with reference to randomly selected features of the same count without shuffling the ages. (B) For comparison and illustration purposes, the shifts in the *r* distributions of observed (orange) and permuted (blue) models fit with only the features within the individual network blocks (as identified as significant in Figure 6C) are plotted with the corresponding Cohen’s *d* values measuring the shift between the observed and permuted distributions. (C) For illustration purposes, these same seven network blocks are compared between prediction accuracy of the individual network block ML models (violin) and the prediction accuracy from ML models based on randomly selected features of the same count as the individual network block (scatter). The “combined” violin plot is the ML model fit with the combined features from all the seven network blocks.

In addition to NLA, we also performed the more standard individual network block analysis, which only uses features from within a single network block for each model and has been employed in previous studies for biological interpretation (Nielsen et al., 2019; Rudolph et al., 2018). We observed that almost all the network blocks were significantly predictive of age when compared to a null distribution generated by shuffling the ages using either FWER-controlled *p* values or Cohen’s *d* (Figure 8A). Since most network blocks were significant using this method (with FWER-controlled *p* values < 0.01/91 or Cohen’s *d* > 0.8), we selected a subset of network blocks based on the significant LSVR results from the NLA model for illustration purposes. Specifically, Figure 8B illustrates the large shifts in the distributions of the Pearson *r* correlation between actual and predicted ages when we compared the observed and permuted models. While the difference in Pearson *r* correlations between actual and predicted ages appeared highly significant, when we instead compared the actual *r* correlations within a network block to actual *r* correlations from an equal count of randomly selected features outside the network block, we observed that there were no network blocks that predicted age better than randomly selected features (Figure 8A). For illustration purposes, we again selected the same subset of seven network blocks and demonstrate the overlap between network-block prediction accuracy (violin) and prediction accuracy from the same number of features randomly selected outside that network block from the rest of the connectome (scatter) (Figure 8C).

## DISCUSSION

This study presents a novel NLA method to facilitate biological interpretability of ML models applied to neuroscience data. We demonstrated that biological interpretations of ML models can be accessed by integrating the NLA method and ML models with inversion, while ML models fit within a single network block were not better than randomly selected features at predicting age. To ensure the effectiveness of this ML+NLA pipeline, we addressed two specific challenges for implementation: (1) we demonstrated the importance of accounting for shared variance from related individuals, and (2) we explored the effect of *k*-best feature selection with Pearson correlation. We observed that ignoring the shared variance resulted in falsely inflated prediction accuracy, while marginal Pearson feature selection resulted in lower prediction accuracy and test-retest reliability.

### Separate Related Individuals Into Training or Test Sets But Do Not Allow in Both

A rising number of researchers are applying ML models to predict behavioral or clinical outcomes using the functional connectome. However, few prior studies have examined standardized methods for modeling shared variance among families (Cohen et al., 2020; Elliott et al., 2019; Feilong et al., 2021). One issue introduced by cross-validation procedures in datasets with related individuals is that the related participants could appear in both the training set and holdout test set. Given that related individuals and twins have connectome data that are more similar than between unrelated strangers (Demeter et al., 2020; Miranda-Dominguez et al., 2018), the shared variance among families violates the assumption that model validation is performed on an independent dataset. As a direct consequence, when predicting the response outcomes in the test set, since there are related participants from the training set whose predictive outcomes are known, the prediction accuracy yielded by the test set cannot be used as a reliable measure for the model performance. In other words, models that fails to account for dependency in participants can lead to an overoptimistic estimate of the performance in the validation set. Therefore, we applied a random sampling regime considering family structure to avoid this drawback. While prediction accuracy and reliability were lower when keeping the participants from the same family together within either training or test set, we can better guarantee that the prediction in the holdout test set is not contaminated by the information in the training set, and therefore prevented the falsely inflated prediction accuracy. Our results have implications for ML analysis of existing datasets with family members such as HCP, ABCD, IBIS, Dominantly Inherited Alzheimer Network, and so forth. And individuals who analyze these datasets should use a cross-validation approach keeping families separated to either train or test datasets. Alternatively, if the goal is to assess the effects of genetic variability on rsFC rather than controlling for it in the model, then traditional regression models such as the generalized linear model (GLM) can be used to estimate heritability from twins by transforming data to squared twin-pair differences (Chen et al., 2019).

### Univariate K-Best Feature Selection With Pearson Correlation Can Lead to Low Prediction Accuracy and Reliability in the Presence of Strong Correlation Among Regressors

In prior ML research, various feature selection methods have been applied ahead of the regression step due to the computational cost of high-dimensional data (Dadi et al., 2019; Nielsen et al., 2019; Shi et al., 2021). Among these methods, the marginal Pearson correlation is a popular option in functional connectome research (Fan et al., 2006; Nielsen et al., 2019). However, based on our study, we found that this additional univariate screening step reduces model performance. Specifically, reducing features via marginal screening methods results in lower prediction accuracy and reliability and different networks associated with outcome prediction or, in the current study, age. This is not simply due to the decrease in the number of features entering the model since the accuracy of prediction tends to converge when the number of features exceeds 1,000 (Nielsen et al., 2019). In particular, they showed that the trace of the prediction accuracy converges exponentially fast when the number of features increases, and the accuracy was not substaintially enhanced when the feature size exceeds 1,000. The suboptimal performance of univariate feature selection methods, such as marginal Pearson correlation, can be attributed to their tendency to select a multitude of strong rsFC features that may arise from redundant correlations among features. For instance, if the connectivity between two networks is correlated with age, the Pearson feature selection will tend to pull many features within that network block. Hence, the information about the relationship between rsFC and age is largely redundant across features within a network block (i.e., all rsFC tend to be positive or all negatively correlated with age to varying degrees).

In contrast, multivariable approaches, like utilizing inversed feature weights from a predictive model such as LSVR, have the advantage of selecting unique and impactful rsFC features that contribute significantly to the prediction, irrespective of correlations among features. Consequently, when relying solely on univariate correlations to select features, those features that could have been crucial for prediction are likely to be eliminated during marginal screenings (Wang et al., 2021). Additionally, multicollinearity can be another main reason for the discrepancies between univariate correlations and LSVR model weights. LSVR tends to allocate its weights among groups of highly collinear features. Therefore, employing “leave-one-feature-out” and “leave-one-feature-group-out” methods can serve as effective alternatives to univariate feature selection (Feng et al., 2013; Guyon & Elisseeff, 2003; Peng et al., 2005).

In our study, we observed that the significant network blocks chosen by Pearson feature selection + LSVR appeared to be more similar to the ones by simple Pearson correlation than they did to the ones by LSVR models. The Pearson feature selection tends to select many redundant features that are correlated among themselves but not necessarily significant for prediction to enter the LSVR model. Also, the redundant features for the ML model are often located within the network blocks. These redundant features would have been removed during the regularization with L^2^-penalty in the full LSVR model. Alternative feature selection methods that account for multivariable relationships between features such as the iterated sure independent screening (Fan & Lv, 2008), and the correlation measures including tilted correlation (Cho & Fryzlewicz, 2012) and quantile partial correlation (Ma et al., 2017) may have greater utility in human connectome analysis.

Furthermore, brain connectivity studies often involve a large number of features (rsFC), which can lead to heavy computational cost. Feature selection becomes crucial in this context, which helps to improve the computational efficiency of the regression model fitting by reducing the number of features entering the model. Therefore, though the feature selection using marginal Pearson correlation does not perform well, it is still worth exploring more effective feature selection techniques and applying them to brain-behavior prediction. Similar to prior research, we also demonstrated that the prediction accuracy can be a function of feature size where the *r* correlation between the predicted and actual labels asymptotically increases with the increasing feature count (Kong et al., 2023; Nielsen et al., 2019). However, this result may not hold for some feature selection methods and datasets, and therefore deserves more investigation in future research. The number of best features should also be carefully chosen in different scenarios.

### NLA on the Inverted Weights Rather Than the Raw ML Weights Provides an Accurate and Biologically Interpretable Framework for Predictive ML Models

The performances of LSVR models without *k*-best feature selection using Pearson correlation were consistent with the existing age prediction literature in both prediction accuracy and reliability (Cui & Gong, 2018; Taxali et al., 2021). Even after correcting the falsely inflated effect caused by the shared variance, our ML model still had competitive prediction accuracy and reliability. We further applied NLA software to determine which network blocks were more associated with age than the rest of the connectome. NLA methods were previously developed for univariate FC association studies (Eggebrecht et al., 2017; Wheelock et al., 2018, 2019, 2021). Here for the first time, we demonstrate their utility in a multivariate setting. Regarding the contribution to the prediction of age, NLA revealed that LSVR coefficients were consistently (across days by test-retest model) stronger and more clustered within several networks including medial parietal (MP), ventral attention (VAN), dorsal attention (DAN), and fronto-parietal (FPN) relative to the rest of brain. Similar to previous literature exploring age associations using linear models and massive univariate analysis (Rieck et al., 2021), we observed a decrease in FC within executive control networks as age increased, which aligns with our findings presented above. Furthermore, consistent with prior research focusing on the network blocks that are most predictive of age using ML models (Dosenbach et al., 2010; Rieck et al., 2021), we also identified the right anterior prefrontal cortex as one of the regions with the highest relative prediction power for age. This region is known to play a crucial role in cognitive control and higher order executive functions. In terms of the reliability of our network-level observations, it is noteworthy that the utilization of MCC scores not only enhances the biological interpretability of our findings, but also yields trustworthy estimates of network-level significance.

Furthermore, concerning the reproducibility of NLA results across three different input sets (i.e., Pearson, Pearson + LSVR, LSVR), we observed some similarities such as medial parietal (MP), ventral attention (VAN), visual (VIS), and ventral attention–medial parietal (VAN-MP) that were implicated in all three methods and two scan days as well as the test-retest. However, considering the similarities between the results by Pearson and Pearson + LSVR, LSVR identified a distinctly different pattern of networks. The possible reason is that the LSVR model tends to put more weight on the features with higher deviation across participants for better prediction accuracy, which yields an optimal combination of features for the purpose of prediction (James et al., 2021). In contrast, Pearson and Pearson + LSVR models selected rsFC features (and by definition the network blocks) with higher individual rsFC relation to the predictive outcome given the massively univariate method of analysis.

To achieve a compromise between the two types of interpretation (i.e., univariate correlation and multivariate regression), we applied the inversion model to the ML pipeline by evaluating the covariance between the rsFC and predicted age obtained from the LSVR model. We observed that the inversion model removed the significant network blocks selected by LSVR due to the high variance across participants, including dorsal attention (DAN), frontal-parietal–default mode (FNP-DMN) and frontal-parietal–dorsal attention (FNP-DAN), while retaining the network blocks selected by univariate type of methods such as auditory-visual (AUD-VIS) and cingulo-opercular–visual (CO-VIS). From the ROI-level heatmaps, it is more explicit that the inversion model yields a similar significance distribution as the Pearson correlation, but with more concentration on VAN, VIS, AUD-VIS, and CO-VIS.

We acknowledge that the MCC score for the LSVR model is marginally higher compared to the LSVR model with inversion, despite both exhibiting a strong positive correlation. Nevertheless, it is important to distinguish between prediction and biological interpretation as distinct objectives. The weights of rsFC features obtained directly from the predictive LSVR model solely reflect the impact of rsFC on age prediction and do not possess any inherent biological significance. Therefore, we highlight that the inversion model provides a more precise biological interpretation by preserving the individual effects of each feature while also considering the full model since the computation of predicted outcomes involved all the features (Haufe et al., 2014). This inversion step is crucial for any future research with a goal of biological ML model interpretation rather than prediction accuracy. Alternatively, for the purpose of interpretation, one can consider employing other models that prioritize interpretability, such as the GLM. Unlike the LSVR models, which often rely on multivariable analysis, a GLM is typically referred to as massively univariate in the sense that it is estimating the effect of age or other covariates at each individual rsFC feature, while a multivariable ML model is fitting age using all the rsFC features as regressors simultaneously. We clarify that multivariate models always focus on relationships between multiple dependent variables by allowing multiple response variables simultaneously (e.g., age and other behavioral scores), taking into account the interdependencies and relationships between them. In contrast, multivariable models predict a single outcome (e.g., age) via multiple predictor variables. In this study, we mainly compared the multivariable models (e.g., LSVR) to the univariate ones (e.g., Pearson feature selection). We observed that multivariable approaches allow for the examination of individual variables while considering their relationship with the outcome of interest, thereby expanding the options available for conducting interpretive analyses.

### NLA on Full Connectome Model Weights is Recommended for Biological Interpretation Rather Than Prediction-Oriented Individual Network Block Analysis

In addition to applying NLA to investigate the biological associations between network blocks and age, we also utilized the prediction-oriented methods introduced in the existing literature (Millar et al., 2020, 2022; Nielsen et al., 2019; Rudolph et al., 2018), where they fit the predictive ML models inside a certain network block and tested the significance of this block by evaluating the prediction shifts between the Pearson *r* distribution of the observed and null models. Consistent with prior research (Nielsen et al., 2019), we found that although the distribution of *r* correlations between actual and estimated age for each significant network block was significantly shifted compared to the permuted null, these network-level models performed no better than models created from an equal number of randomly selected features from the rest of the connectome. This suggests that ML models generated on each individual network block cannot be used to infer biological specificity of associations with behavior or clinical outcomes.

In contrast, we emphasize that the significant network blocks identified using NLA have been determined with respect to features from the whole connectome. We did not compare the *r* distributions of the random model and the predictive model fit by the features selected by NLA because *r* correlation is a measure of prediction accuracy while NLA performs statistical inference. However, where individual network block analysis is not able to exceed the performance of randomly selected rsFC throughout the rest of the connectome, NLA is able to identify the associations within a network block that exceed the expected rate based on all other features in the rest of the connectome.

Furthermore, we note that, alternative ML approaches have been developed to address the loss in accuracy from reduced feature sets. Specifically, the leave-one-feature-group-out technique retrains the model with a brain system (network block) left out, thus retaining thousands of features from the connectome in the model. The loss in predictive performance is considered as a proxy for the importance of that network block (Feng et al., 2013; Guyon & Elisseeff, 2003; Nielsen et al., 2023; Peng et al., 2005). To ascertain whether the impact of the left-out rsFC features in a specific network block was due to the unique identity of this block rather than the number of features it contains, the impact of the same number of randomly left-out rsFC features should also be examined as a null model (Nielsen et al., 2023). As such, the drawback of the prediction-oriented ML approaches can be properly addressed. However, it is also worth investigating the reliability of this type of approach relative to NLA, and we will address this aspect in our future work.

In summary, we point out that attempting to model individual network blocks in isolation would fail to provide biological interpretations of the ML results for three reasons: (1) individual network block analysis does not result in prediction accuracy outperforming randomly selected features (Nielsen et al., 2019), (2) the networks with higher prediction accuracy may not be biologically interpretable because the most predictive features without inversion may be due to nonneuronal sources of variation (Haufe et al., 2014), and (3) prediction accuracy is dependent on the size of the network, making different networks incomparable if modeled individually (Millar et al., 2022; Nielsen et al., 2019). Consequently, we highlight that ML models utilizing whole-connectome data with the hypothesis testing framework of NLA provides a more robust avenue for biological interpretation of ML modeling, instead of fitting prediction-oriented models on the features within each individual network block.

### NLA Goes Beyond a Feature Reduction Step and Switches the Focus From Prediction to Interpretation

The proposed NLA employs enrichment analysis and can be used to identify brain system associations with outcome variables by inferential statistics. However, we emphasize that NLA is not a feature selection step. Rather, NLA reduces the number of multiple comparisons for statistical inference by assessing system-level associations with outcome variables. NLA leverages canonical atlases of system-level connectivity that acts not only as a data reduction step, but provides a biological framework in which to interpret associations with outcomes. In contrast, the focus of traditional feature selection methods such as PCA/ICA and LASSO lies in optimal predictive performance, which means the estimated weights are intermediate steps toward a good predicted outcome, not the primary purpose. Therefore, the significant features selected by these traditional feature selection methods can lead to inaccurate conclusions regarding the spatial or temporal origin of the neural signals of interest. For example, the rsFC features with significant nonzero weights may be observed; however, these features can be statistically independent of the brain process under study (Chen et al., 2022; Haufe et al., 2014). In addition, dimension reduction techniques including PCA/ICA essentially adopt principal features in a different feature space for model fitting, which can be hard to map back to the original rsFC features in brain. Even if one is able to map back the principal component to rsFC features, there might be limited discernable biological pattern to these rsFC features, which can spread across the brain or among several different brain systems (Seitzman et al., 2019; Wig, 2017). To summarize, NLA provides a biological interpretation framework for ML models, specifically by identifying network blocks of rsFC features that meet the connectome-wide FWER significance criteria. NLA serves a different purpose (interpretation-oriented) compared to traditional ML methods (prediction-oriented).

### Limitations and Future Directions

One limitation of this paper is that all analyses were validated on the HCP dataset, and it is crucial to extend our analysis to other datasets to obtain a more comprehensive scope and impact of our findings. Specifically, the range of age in the current study is limited, and researchers seeking to understand the biological basis of aging should apply network-level enrichment to datasets containing a wider range of ages. In addition, the Pearson correlations we used as connectivity features can be replaced by the regularized partial correlations, which recently have been shown to enhance the model performance by a number of studies (Epskamp & Fried, 2018; Peterson et al., 2023). Furthermore, considering the imperfectness of marginal feature selection, alternative feature selection methods should be investigated that incorporate multivariable feature selection. It is also worth noting that unlike the univariate Pearson correlation, predetermining the number of selected features to be 1,000 might not be enough to achieve desired predictive performance using multivariable feature selection methods. The convergence of prediction accuracy needs further investigation for more advanced feature selection methods. Finally, we only focused on the LSVR model in this study because we were iterating on many other methods for implementation and interpretation. However, many different regression models have been used in connectome data that have shown promise, such as canonical correlation analysis (Mihalik et al., 2022), deep artificial neural networks (He et al., 2020; Niu et al., 2020), random forest (Cohen et al., 2020; Jollans et al., 2019), and so forth. Additional work is needed to validate NLA with these alternative ML models and to determine how these models and corresponding feature selection methods impact biological interpretation of prediction weights.

## CONCLUSION

The present study proposed a network-level enrichment to facilitate biological interpretability of ML models. For the implementation of the ML+NLA pipeline, we have provided practical guidelines, including a resampling technique accounting for related participants such as siblings and family members. In the presence of complex correlation structure among the regressors, feature selection approaches based on marginal correlation (e.g., Pearson) could preserve redundant features and rule out the predictive features that should have been useful together with other features, thus harming the model performance. Unlike previous studies on ML models that primarily concentrated on predictive accuracy, the importance of evaluating the distinct contributions of each brain network should not be overlooked. This can be effectively achieved by integrating ML with comprehensive connectome data, and applying NLA to the inversion model, thereby ensuring thorough and reliable biological interpretations.

## CITATION DIVERSITY STATEMENT

Recent work in neuroscience has identified a bias in citation practices such that manuscripts written by women and other minorities are undercited relative to the number of such papers in the field (Dworkin et al., 2020). Here we quantify the citation diversity of the present manuscript excluding self-citations of the first and last authors of this manuscript. Our reference list contains 52% man-man (first author–last author), 14% woman-man, 22% man-woman, and 12% woman-woman citations.

## SUPPORTING INFORMATION

Supporting information for this article is available at https://doi.org/10.1162/netn_a_00383. Human Connectome Project data are available at https://db.humanconnectome.org/. Network-level analysis code available at https://github.com/WheelockLab/NetworkLevelAnalysisBeta and https://github.com/WheelockLab/MachineLearning_NetworkLevelAnalysis.

## AUTHOR CONTRIBUTIONS

Jiaqi Li: Conceptualization; Formal analysis; Investigation; Methodology; Project administration; Software; Validation; Visualization; Writing – original draft; Writing – review & editing. Ari Segel: Formal analysis; Visualization; Writing – review & editing. Xinyang Feng: Formal analysis; Writing – review & editing. Jiaxin Cindy Tu: Data curation; Resources; Writing – review & editing. Andy Eck: Software; Writing – review & editing. Kelsey King: Data curation; Resources; Writing – review & editing. Babatunde Adeyemo: Data curation; Writing – review & editing. Nicole R. Karcher: Data curation; Writing – review & editing. Likai Chen: Writing – review & editing. Adam Eggebrecht: Conceptualization; Writing – review & editing. Muriah Wheelock: Conceptualization; Data curation; Formal analysis; Funding acquisition; Investigation; Methodology; Project administration; Resources; Software; Supervision; Validation; Visualization; Writing – original draft; Writing – review & editing.

## FUNDING INFORMATION

Muriah Wheelock, National Institute of Biomedical Imaging and Bioengineering (https://dx.doi.org/10.13039/100000070), Award ID: EB029343.

## Supplementary Material



## TECHNICAL TERMS

Raw model weight: Estimated coefficient of a feature (i.e., a predictor or regressor) from a fit regression model.
Inversion model: Models using the covariance between a feature (i.e., a predictor or regressor) and the predicted outcome as the estimated coefficient.
Network block: A block of functional connectivities, where the block is defined by network-level architecture of brain as described in several canonical atlases.
Network level analysis (NLA): An enrichment analysis integrating brain system organization with connectome-wide statistical analysis to reveal network-level links between brain connectivity and behavior.
*K*-best feature selection: A feature selection method that selects the top-ranking K many features according to some given rule (e.g., Pearson correlation).
Inner cross-validation (CV): Another cross-validation embedded within the training set of each resampling repetition, typically for the purpose of hyperparameter tuning.
Permuted model: A regression model fit on a set of randomly shuffled outcome labels.
Individual network-block analysis: A significance test that only uses rsFC within a single network block to generate r-correlations between predicted and true outcomes.
Multivariate model: A model focuses on relationships between multiple dependent variables by allowing multiple response variables simultaneously (e.g., age and other behavioral scores).
Multivariable model: A model predicts a single outcome (e.g., age) via multiple predictor variables.
