# Supplementary material for: Network-level enrichment provides a framework for biological interpretation of machine learning results
Source: Netw Neurosci. 2024 Oct 1;8(3):762–90. doi: 10.1162/netn_a_00383 (PMC11349033; doi:10.1162/netn_a_00383)
Supplement: Supplementary file 1 [file netn-8-3-762-s001.pdf]

Supplementary Materials

Linear Support Regression Model

For n=965 participants, we observed the ages $y_1, y_2, \ldots, y_n$, and the feature vectors $X_1, X_2, \ldots, X_p$, where $X_j = \left(x_{1,j}, x_{2,j}, \ldots, x_{n,j}\right)^T$ with $x_{i,j}$ being the observed j-th rsFC for the i-th subject. In each random sampling round, we assigned 80% family to the training set, and fit the linear regression model as follows:

$$\hat{y} = \sum_{j=1}^{p} \hat{\beta}_j X_j + \hat{\beta}_0,$$

where the vector $\hat{y} = \left(\hat{y}_1, \hat{y}_2, \ldots, \hat{y}_{n\_train}\right)^T$ consists of predictive labels (i.e., age) of all the family members in the training set, and $\hat{\beta}_j$ is the estimated beta weight for the j-th feature in rsFC. Our goal is to train the above ML regression model and find optimal $\hat{\beta}_j$, $\mathrm{j} = 1, 2, \ldots, 55278$, and $\hat{\beta}_0$ such that this model could best predict the actual ages $y_i, i = 1, \ldots, 965$. Since the number of features exceeds the sample size, we applied the Linear Support Vector Regression (LSVR) to deal with the high-dimensional problem and estimated these regression coefficients. Specifically, we applied a Vapnik's ϵ-sensitive loss function (Vapnik 1999) to fit the above linear model. Define $f(X_i) = \sum_{j=1}^{p} \beta_j x_{i,j} + \beta_0$. We aim to minimize the objective function below

$$\min_{\beta} \frac{1}{2} \sum_{j=1}^{p} \left|\left|\beta_j\right|\right|^2 + \lambda \sum_{s=1}^{l} (\xi_s + \xi_s^*)$$

$$subject\ to \begin{cases} y_i - f(X_i) \leq \epsilon + \xi_i \\ f(X_i) - y_i \leq \epsilon + \ \xi_i^* \\ \xi_i,\ \xi_i^* \geq 0 \end{cases}$$

where l is the number of "support vectors", which can be interpreted as the number of samples that deviate from the true y_i by more than ϵ. The hyper-parameter λ controls the trade-off of

penalties between the bias and variance. In particular, a large λ gives more penalties on bias, and a small λ assigns more penalties to variance. In this study, we used a nested cross-validation to choose the optimal λ. Please see the section below for details.

Cross-validation for tuning parameters

Note that to handle the dimension issue, the LSVR model incorporates two parts in the loss function, which are the regularized L2-norm term aggregating all the beta weights and another Vapnik's ε-loss function. We follow the standard construction of these two terms as applied in a previous study (Cui and Gong 2018) and shall save the details here due to the relevance. However, a tuning parameter balancing these two terms is not trivial to derive. In order to find the optimal tuning parameter, we inserted a nested 5-fold cross-validation in each 1000 random sampling repetition. Specifically, for each outer-sampling round, we randomly assigned 80% of families to the training set and the remaining 20% to the test set. For each nested 5-fold cross-validation, we again subsampled the training set into an 80% training set and a 20% test set to find an optimal tuning parameter by minimizing the loss function mentioned above. This nested 5-fold cross-validation is shown in Figure S1.

In each nested 5-fold cross-validation subsampling repetition, we adopted a grid search for λ with the range between 10^{-5} to 10^{-1}. There were 15 searched points on the grid, which are $1.000*10^{-5}$, $1.931*10^{-5}$, $3.728*10^{-5}$, $1.389*10^{-4}$, $2.683*10^{-4}$, $5.179*10^{-4}$, $1.000*10^{-3}$, 0.0019, 0.0037, 0.0072, 0.0139, 0.0268, 0.0518, 0.1000. These searched points were randomly chosen to cover the search range as uniformly as possible, and they remained the same for all 1000 repetitions. The mean of

the optimal λ identified by nested 5-fold cross-validation over 1000 repetitions is 0.0332 with the variance 0.0365.

Prediction accuracy of ML approaches

Aside from the correlation between the predicted labels and the actual ones, we provide two additional commonly used measures, Mean Absolute Error (MAE) and Mean Square Error (MSE) (He et al. 2020; Niu et al. 2020; Modabbernia et al. 2021) to evaluate the prediction accuracy of four ML approaches with different feature filters applied ahead of different random sampling methods utilized for the regression model fitting. Table S1 shows the MAE and Table S2 presents the MSE, from which we could draw two conclusions: (1) if the shared variance among siblings/families were neglected, then the prediction accuracy of the ML model would be falsely inflated; (2) Marginal Pearson filter applied ahead of the ML model lowers the prediction accuracy.

Corrected resampling t-test

Here we show the details of modified t-test to account for the issues of subsampling and multiple comparison. Let n_1 be the number of participants in the training set (e.g., 965*0.8) and n_2 be the number of participants in the test set (e.g., 965*0.2). We performed N=1000 repetitions of subsampling. For the k-th repetition, suppose the difference between the prediction accuracies (e.g., MAE) of Model A and Model B is d_k. Then, compute the mean of difference $\bar{d} = \sum_{k=1}^{N} d_k / N$ and the variance $\sigma^2 = \sum_{k=1}^{N} \frac{(d_k - \bar{d})^2}{N-1}$. The corrected variance is $\sigma_{mod}^2 = \left(\frac{1}{N} + \frac{n_1}{n_2}\right) \sigma^2$ and the corrected t-test statistic is $\bar{d}/\sigma_{mod}$.

FWER-controlled p-values

In the permutation test introduced in Section 2.5.2, once the $\chi 2$ test statistics were computed for observed and permuted weight matrices, we computed permutation-based p-values by performing a familywise error rate correction (FWER) control. We consider the full connectome as a family and ranked the χ^2 test statistics of the observed network compared to all the 1000*91 (repetitions * network blocks) permuted χ^2 test statistics. Specifically, all the χ^2 test statistics were concatenated together and then ranked from smallest to largest. The rank of the observed χ^2 test statistics, number of permutations, and number of network blocks were used to calculate an experiment wide permutation-based, network level p-value. We define this FWER-controlled p-value as $\mathrm{p} = (1 + \mathrm{rank})/(1 + 1000 * 91)$, where 1000 is the number of permutations and 91 is the total number of network blocks. For example, if the observed χ^2 statistic of the network block "VAN-MP" is 5.61, and among the 1000*91 permuted χ^2 statistics, only 800 of them are larger than 5.61, then the rank of "VAN-MP" is 800 and the FWER-controlled p-value is ~0.009.

Different thresholds for Z-scores in NLA

As mentioned in Section 2.5.1, we took the Z-scores of the weight matrices and thresholded the Z-scores by $|Z|>2$ after experimenting with several thresholds. See for example, in Figure S2, we presented $|Z|>2.5$, $|Z|>2$ and $|Z|>1.5$ for the four model: (1) Pearson, (2) Pearson + LSVR, (3) LSVR, and (4) LSVR + inversion. We can clearly see from the edge-level results in Figure S2(A) that the lower threshold has more "pepper" compared to the thresholds 2 and 2.5. In contrast, the network level results are relatively similar across the three thresholds. To see this, for each model,

we quantified the MCC scores of "|Z|>1.5 vs. |Z|>2", and "|Z|>2.5 vs |Z|>2", by treating the significant network blocks in |Z|>2 as TP, the remaining blocks as TN. The blocks that are significant in |Z|>1.5 (or>2.5) but not in |Z|>2 were treated as FP. Similarly, the blocks that are insignificant in |Z|>1.5 (or>2.5) but not in |Z|>2 were treated as FN. The MCC scores are reported in Figure S2(B), which are all exceeding 0.7.

However, it is still worth noting that different thresholds can alter the interpretation of data. Alternative NLA methods that do not rely on thresholding and binarization are in development by the authors. In this study, we stick with the threshold |Z|>2 for two reasons. (1) The edge-level result by the "Pearson + LSVR" model was already sparse. Thus, for the sake of comparison between different models, a z threshold was applied across all methods; (2) A network level statistic that relied on unthresholded and unbinarized data would not be as effective as the model weights within a network block that demonstrated high magnitudes in opposite directions (i.e., a mix of negative and positive weights).

Network permutation

In the permutation test conducted for the network-level analysis, we randomly shuffled the ages across participants and then estimated the weights of rsFC features through these null models. Prior research suggests that permuted models might not be suitable for the null distribution when data do not respect exchangeability under the null hypothesis, such as the tests with known dependence among participants (e.g., family members) and instead a block permutation would be more appropriate (Winkler et al. 2014). Following these guidelines, we randomly shuffled the ages across the families, while still keeping the family members together within either the training set or the test set, i.e., viewing each family as a block in our permutation test. We observed that the permuted weight matrices generated by LSVR models displayed a consistent structural pattern

across multiple sampling repetitions. Interestingly, this pattern closely mirrored the standard deviation observed in the functional connectivity matrices across all participants. This actually mirrors the practice of feature scaling when fitting ML models, that is, normalizing the range of features in the dataset. However, it deserves future study to see how much the scaling of rsFC features would affect the model performance and if this would overlook the underlying structure of brain connectivity matrices.

Furthermore, prior work has suggested that the rsFC features should be permuted in addition to permuting the labels (Xia et al. 2018). We observed that, while a model that shuffles both networks and labels may yield a higher degree of randomness, it risks losing the intrinsic covariance structure of the connectome. Specifically, when we only randomly shuffled ages, the permuted weight matrices still had structured patterns of relatively stronger weights within network blocks that changed location for each permutation. We showed the permuted matrices by these two approaches and compared the patterns. In Figure S3, we list five permuted estimated weight matrices from the ML model with a) labels randomly shuffled, and b) both labels and networks randomly shuffled. For these two implementations, we estimated the weights via three methods: 1) calculating the Pearson correlation between each feature and the age, 2) estimating the regression coefficients by utilizing the LSVR model with no feature filter applied, and 3) calculating the inverse LSVR model weights. We observed that if we interrupted both the feature and label orders, the strength of the weights appeared relatively uniform across the permuted matrices. However, when only permuting the labels without shuffling the networks, a more structured pattern of weights corresponding to the network blocks appeared in each random sampling loop. When the Pearson and the inverted LSVR methods were applied, the location of relatively stronger weights within network blocks differed across each permutation, while the

patterns from the LSVR models (Row 2 in Figure S3) reflect the underlying variance structure of the rsFC for all participants.

Given this observation, we conclude that null models generated by shuffling both networks and predictive values can lead to questionable significance results in subsequent hypothesis testing due to the break of autocorrelation properties in data. In the voxel-wise literature, in the presence of spatial dependence among voxels, random sampling cannot be performed independently voxel by voxel, but rather entire images should be resampled as a whole to preserve such dependence structure (Nichols and Hayasaka 2003). Hence, we recommend future researchers analyzing the connectome only shuffle the behavior or clinical outcome labels and preserve the covariance structure of the connectome for biological realism during permutation testing.

Network Level Enrichment Methodology

(a) Selection of Brain Network Map

Network Level Analysis (NLA) necessitates that researchers choose a specific network map to portray the established connectome architecture. This selection is vital, as it influences both the statistical significance testing and subsequent interpretations. Researchers may select their preferred algorithm for community detection or utilize one of the various published ROI and network maps included in the NLA toolbox. Employing standardized ROI and network maps establishes a consistent and reproducible framework for evaluating brain-behavior relationships within connectome studies.

(b) Statistical Model Selection for General Linear Model / Edge-wise Analysis

The NLA requires researchers to choose an appropriate statistical model to examine the relationships between behavioral data and connectivity data between ROI-pairs (i.e., edge-level). The NLA framework provides options for both parametric and non-parametric correlations, accommodating a range of analytical preferences. We also provide our LSVR scripts which can generate inverted ML weights for use in the NLA framework (see our code on https://github.com/WheelockLab/MachineLearning_NetworkLevelAnalysis).

(c) Generation of Connectivity Matrices

Connectivity matrices, which form the basis for input into the NLA toolbox, can be generated using external software packages. A popular option is CONN, a comprehensive MATLAB-based program which can construct functional connectivity matrices derived from fMRI data.

(d) Procedure of Network-level analysis

Following this, network level analysis (NLA)—including the transformation of edge-wise statistics and enrichment statistic computation—determines which networks have a strong association with the outcome under study. The NLA pipeline supports both continuous edge-level input and sparse (i.e., thresholded and binarized) input.

For computing enrichment statistics, NLA includes various statistical tests. For analysis of thresholded and binarized edge-level data, NLA offers the chi-square test and the hypergeometric network-level tests. Significance is established through data-driven permutation tests, comparing observed enrichment statistics against a null distribution formed by random permutation of the outcome measure labels. In this study, we adopted a chi-square test and a nominal threshold of $|Z|>2$ for the thresholding and binarization step of the edge-level tests. Here, we introduce some necessary definitions and provide the formula to calculate the chi-square statistic.

For an observed weight matrix, we calculated the Z-scores. We call an ROI pair as a "hit" if $|Z|>2$ and set it to be 1, otherwise we call it "non-hit" and set it to be 0. For brevity, we give the following definitions:

- N_hit = total number of hits across the full connectome;
- N_ROI = total number of ROI pairs in the connectome;
- Actual_ hits = number of observed hits within a given network block (NB);
- Expected_hits = number of expected hits within an NB;
- Expected_significance_ratio = N_hit/N_ROI

In fact, the "Expected_hits" is relative to the hit rate in the full connectome, thus we can write "Expected_hits" as "Expected_hits = Expected_significance_ratio * number of ROI pairs in an NB". By computing both "Actual_hits" and "Expected_hits", we can obtain the chi-square statistic

$$\chi^2 = (\text{Actual\_hits} - \text{Expected\_hits})^2 * \left(\frac{1}{\text{Expected\_hits}}\right).$$

We can calculate the p-values via the function "chi2cdf" in MATLAB.

Furthermore, we employ an additional step to control the significant p-values due to depletion, that is, the significant p-values which satisfy "Expected_hits (Es) > Actual_ hits (As)". Specifically, we create a vector corresponding to the number of NB in the connectome. This vector is used to count whether As > Es. For the NB that satisfy As > Es, we set the corresponding entry of the vector to be 1, otherwise to be 0. Then, we apply the vector to the previous p-values calculated from the chi-square test as an exponent. This additional step is important because when As and Es differ, regardless of As > Es or Es > As, $(As\text{-}Es)^2$ will be large, which leads to a large chi-square

statistic, even when As = 0 (i.e., when the observed hits within an NB is 0). By applying this additional step, we can successfully retain the valid enrichment statistics where As > Es only.

Summary of low-motion data and threshold for data inclusion

ML models can pick up on variance due to number of retained minutes of data and spuriously attribute these differences to prediction. For example, if a clinical group moves more than a control group, and on average their participants have fewer minutes of data in the rsFC matrices, then the ML model can pick up on this difference within the rsFC and will boost the prediction of the clinical group labels despite the fact that this is non-neuronal information. Therefore, for ML methods, all participants must have the same number of minutes of data when estimating the rsFC matrix. This leads to a trade-off between minutes of data and number of participants included in the model. We report the range and mean of minutes of available low motion data across participants (Figure S4). Using a 10-minute cutoff allowed us to retain 80% of the HCP sample (N=965) and to have a large enough group to robustly spilt the sample for training and testing. We note that we did not take the first 10 minutes of data but randomly subsampled 10 minutes from the low-motion data. This has been shown to be more reliable than sampling continuous segments of data (Figure 4B in Laumann et al. 2015) because the time series are temporally dependent. Moreover, prior work has shown that reliability and similarity can be greatly improved by increasing the scan lengths from 5 min up to 13 min, and that both the increase in the number of volumes as well as the increase in the length of time over which these volumes was acquired drove this increase in reliability. This improvement in reliability due to scan length is much greater for scans acquired during the same session. Gains in intersession reliability began to diminish after 9–

12 min, while improvements in intrasession reliability plateaued around 12–16 min (Birn et al. 2013).

Prediction bias by motion

In Figure S5, we reported the quality control plots to show the prediction bias by motion. As shown in the figure, the FC-motion correlation is centered around zero (where motion was quantified as mean retained frame displacement). The FC-FD-distance correlation is relatively flat. In particular, the median absolute FC-FD correlation is 0.06 on both Rest day 1 and 2, and the FC-FD-distance correlation is -0.08 on Rest day 1 and -0.06 on Rest day 2. Our FC-motion correlation is comparable to the confounding regression models investigated in (Ciric et al. 2017) (Fig 3B therein), whose best methods yielded FC-correlations around 0.044. Our FC-FD-distance correlation is also comparable to the ones in (Ciric et al. 2017) (Fig 4B therein) whose smallest magnitude is 0.031. Our model ranked between the best two models in their paper.

Table S1 Mean Absolute Error (MAE) of four ML approaches.

MAE	No Filter		Pearson Filter	
	RS1 (Randomly Split)	RS2 (Families Together)	RS1 (Randomly Split)	RS2 (Families Together)
Rest 1	3.1722	3.2391	3.1878	3.2617
Rest 2	3.2221	3.2761	3.2804	3.3560
Test-retest	3.1970	3.2457	3.2657	3.3266

Table S2 Mean Square Error (MSE) of four ML approaches.

MSE	No Filter		Pearson Filter	
	RS1 (Randomly Split)	RS2 (Families Together)	RS1 (Randomly Split)	RS2 (Families Together)
Rest 1	15.2517	15.8611	15.6208	16.3052
Rest 2	15.6774	16.1364	16.5109	17.2508
Test-retest	15.4207	15.8931	16.4068	16.9566

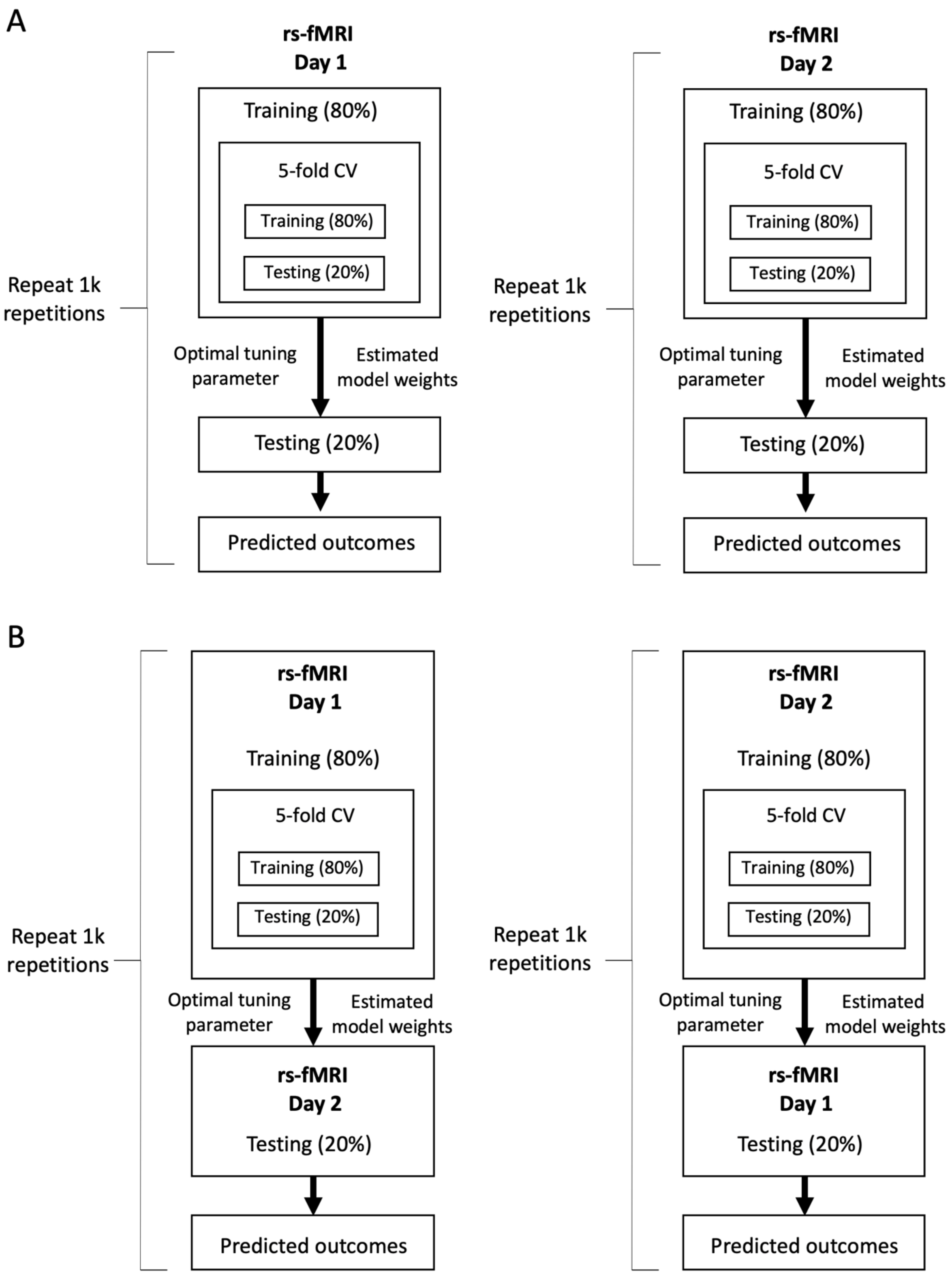

Figure S1. The nested 5-fold cross-validation (CV) for the ML pipeline. (A) The nested 5-

fold CV on two separate rest scan days. **(B)** The nested 5-fold CV for the test-retest.

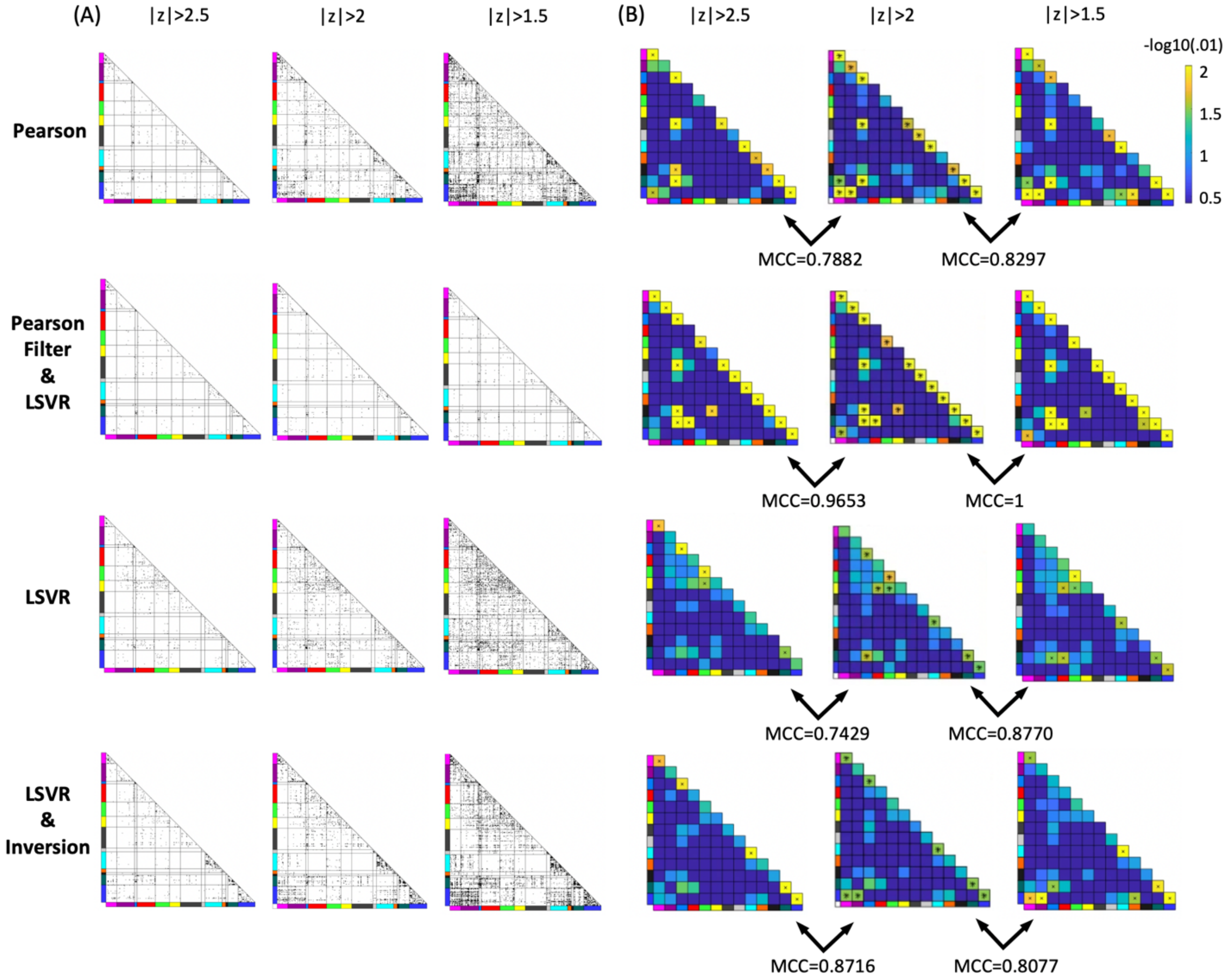

Figure S2. Effects of different thresholds for Z-scores on test-retest data. (A) Significant rsFC features selected by four different methods; **(B)** Significant network blocks selected by NLA with the inputs from four different methods. In each subfigure, three different thresholds of Z-scores are included: (Left) |Z|>2.5; (Middle) |Z|>2; (Right) |Z|>1.5.

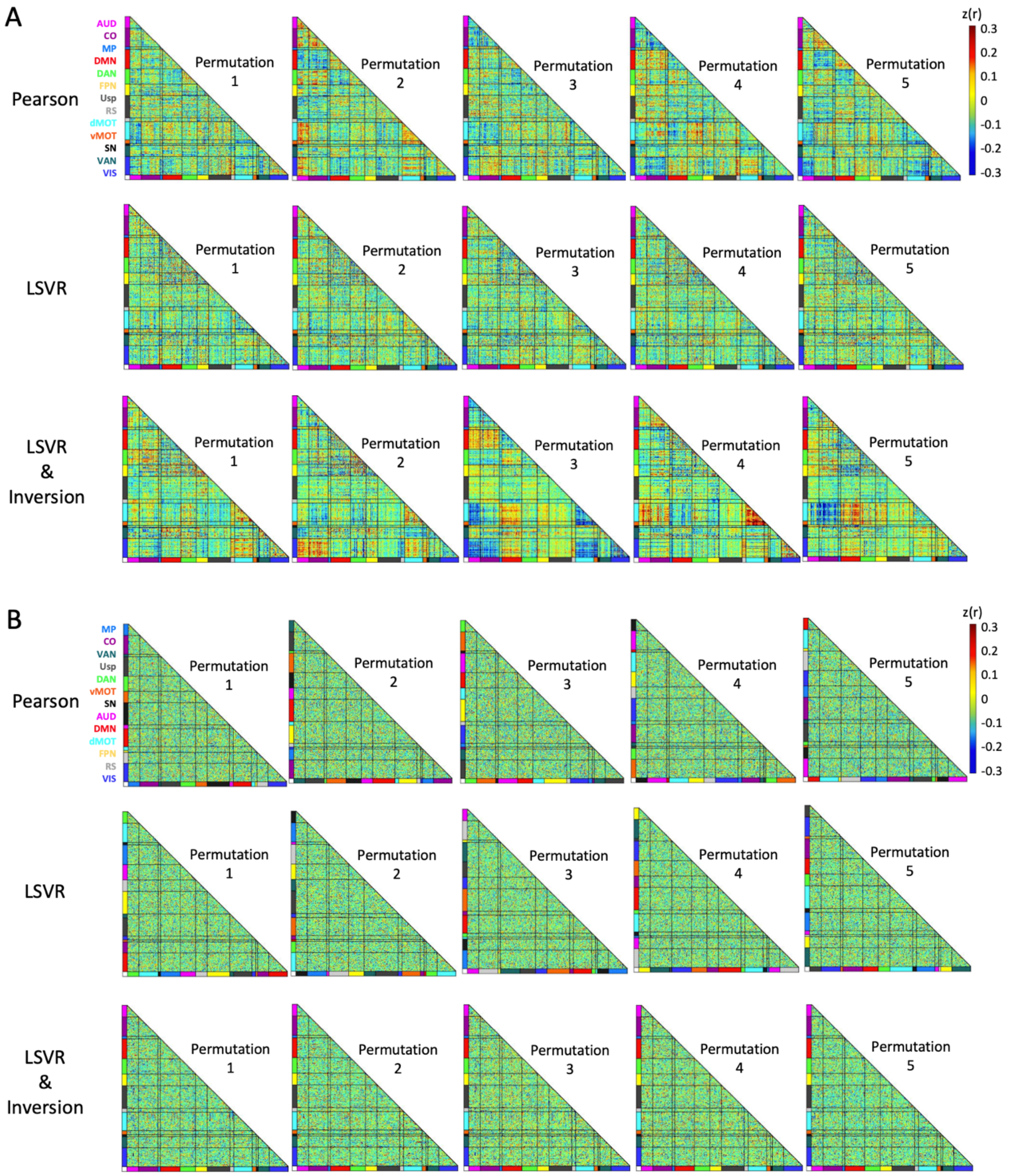

Figure S3. The first five permuted estimated weight matrices from the ML model. We examined three estimation methods: Pearson, LSVR and LSVR with inversion. **A.** Only age labels were randomly shuffled. **B.** Both labels and networks were permuted.

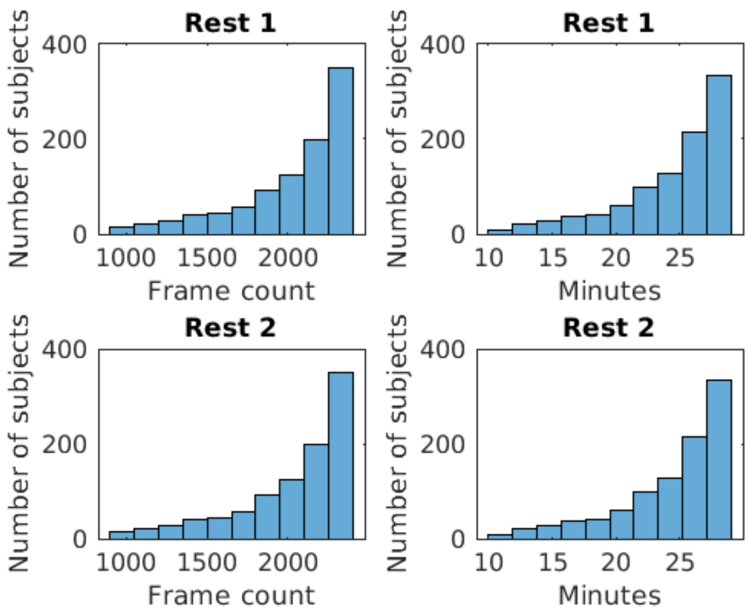

Figure S4. The histogram of the number of frames and minutes acquired from participants on Rest 1 and Rest 2 data. (Left) Distribution of frames; **(Right)** Distribution of minutes.

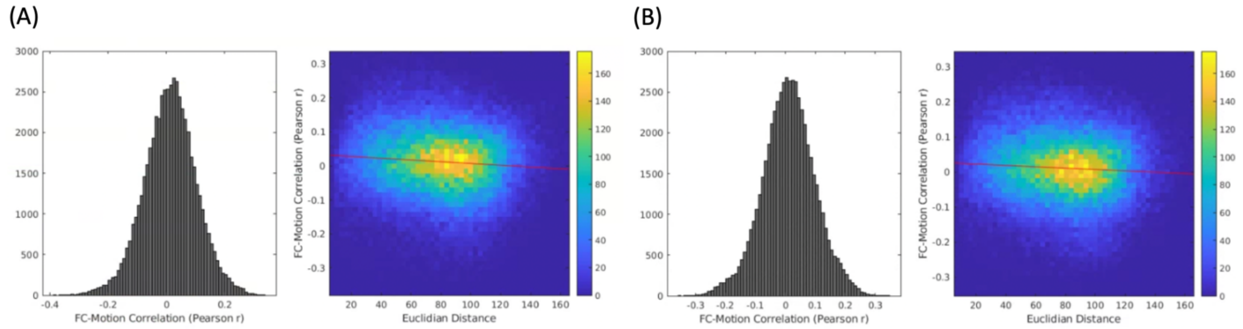

Figure S5. QC plots. (A) Rest Day 1; **(B)** Rest Day 2. In each subfigure, the left panel is FC-FC correlation and the right panel is FC-FD-distance correlation.

References

Birn, Rasmus M., Erin K. Molloy, Rémi Patriat, Taurean Parker, Timothy B. Meier, Gregory R. Kirk, Veena A. Nair, M. Elizabeth Meyerand, and Vivek Prabhakaran. 2013. "The Effect of Scan Length on the Reliability of Resting-State fMRI Connectivity Estimates." *NeuroImage* 83 (December): 550–58. https://doi.org/10.1016/j.neuroimage.2013.05.099.

Ciric, Rastko, Daniel H. Wolf, Jonathan D. Power, David R. Roalf, Graham L. Baum, Kosha Ruparel, Russell T. Shinohara, et al. 2017. "Benchmarking of Participant-Level Confound Regression Strategies for the Control of Motion Artifact in Studies of Functional Connectivity." *NeuroImage* 154 (July): 174–87. https://doi.org/10.1016/j.neuroimage.2017.03.020.

Cui, Zaixu, and Gaolang Gong. 2018. "The Effect of Machine Learning Regression Algorithms and Sample Size on Individualized Behavioral Prediction with Functional Connectivity Features." *NeuroImage* 178 (September): 622–37. https://doi.org/10.1016/j.neuroimage.2018.06.001.

He, Tong, Ru Kong, Avram J. Holmes, Minh Nguyen, Mert R. Sabuncu, Simon B. Eickhoff, Danilo Bzdok, Jiashi Feng, and B. T. Thomas Yeo. 2020. "Deep Neural Networks and Kernel Regression Achieve Comparable Accuracies for Functional Connectivity Prediction of Behavior and Demographics." *NeuroImage* 206 (February): 116276. https://doi.org/10.1016/j.neuroimage.2019.116276.

Laumann, Timothy O., Evan M. Gordon, Babatunde Adeyemo, Abraham Z. Snyder, Sung Jun Joo, Mei-Yen Chen, Adrian W. Gilmore, et al. 2015. "Functional System and Areal Organization of a Highly Sampled Individual Human Brain." *Neuron* 87 (3): 657–70. https://doi.org/10.1016/j.neuron.2015.06.037.

Modabbernia, Amirhossein, Heather C. Whalley, David C. Glahn, Paul M. Thompson, Rene S. Kahn, and Sophia Frangou. 2021. "Systematic Evaluation of Machine Learning Algorithms for Neuroanatomically-Based Age Prediction in Youth." bioRxiv. https://doi.org/10.1101/2021.11.24.469888.

Nichols, Thomas, and Satoru Hayasaka. 2003. "Controlling the Familywise Error Rate in Functional Neuroimaging: A Comparative Review." *Statistical Methods in Medical Research*. https://doi.org/10.1191/0962280203sm341ra.

Niu, Xin, Fengqing Zhang, John Kounios, and Hualou Liang. 2020. "Improved Prediction of Brain Age Using Multimodal Neuroimaging Data." *Human Brain Mapping* 41 (6): 1626–43. https://doi.org/10.1002/hbm.24899.

Vapnik, Vladimir. 1999. *The Nature of Statistical Learning Theory*. Springer Science & Business Media.

Winkler, Anderson M., Gerard R. Ridgway, Matthew A. Webster, Stephen M. Smith, and Thomas E. Nichols. 2014. "Permutation Inference for the General Linear Model." *NeuroImage* 92 (May): 381–97. https://doi.org/10.1016/j.neuroimage.2014.01.060.

Xia, Cedric Huchuan, Zongming Ma, Rastko Ciric, Shi Gu, Richard F. Betzel, Antonia N. Kaczkurkin, Monica E. Calkins, et al. 2018. "Linked Dimensions of Psychopathology and Connectivity in Functional Brain Networks." *Nature Communications* 9 (1): 3003. https://doi.org/10.1038/s41467-018-05317-y.
